# Money and happiness: the income–happiness correlation is higher when income inequality is higher

**DOI:** 10.1093/pnasnexus/pgac224

**Published:** 2022-10-08

**Authors:** Shigehiro Oishi, Youngjae Cha, Asuka Komiya, Hiroshi Ono

**Affiliations:** Department of Psychology, University of Chicago, Chicago, IL 60637, USA; Department of Psychology, University of Virginia, Charlottesville, VA 22904-4132, USA; Graduate School of Integrated Arts and Sciences, Hiroshima University, Hiroshima 739-8511, Japan; School of International Corporate Strategy, Hitotsubashi University Business School, Tokyo 101-8439, Japan

**Keywords:** happiness, income inequality, income, culture

## Abstract

Has the income–happiness correlation changed over time? If so, what predicts such changes? We tested these questions in diverse economic, political, and cultural contexts. Drawing on nationally representative data, we found that the income–happiness correlation has increased in the USA since 1972, as GDP per capita and income inequality increased (Study 1). Study 2 examined an income–life satisfaction correlation in nationally representative surveys between 1978 and 2011 in Japan. Unlike in the USA, there was no clear increase in the income–life satisfaction correlation over time. We next examined the income–life satisfaction correlations in 16 European countries and found that on average the income–life satisfaction correlation has increased since 1970, and it was particularly high in years of high GDP per capita and high-income inequality (Study 3). Finally, we found that among Latin American countries, the income–life satisfaction correlation has, on average, decreased since 1997, as income inequality has decreased (Study 4). Over the last 5 decades, the income–happiness correlation has increased, not decreased, in the USA and several European countries. The income–happiness correlation tends to get higher when both GDP per capita and income inequality are high, whereas it tends to get lower when GDP per capita and/or income inequality are low. These findings suggest the importance of accounting for income inequality as well as national wealth in understanding the role of money in happiness.

Significance Statement Past research examined an income–happiness correlation in one cultural context over one time period, limiting our knowledge regarding whether the nature of the income–happiness correlation has changed over a long period of time, and if so, what predicts such changes. We found that the income–happiness correlation has increased in the USA and Europe since the 1970s, as both GDP per capita and income inequality increased. In contrast, the income–life satisfaction correlation has decreased since 1997 among Latin American countries, where income inequality has decreased during that time. The income–happiness correlation tends to get higher when GDP per capita and income inequality are high while getting smaller as GDP per capita and income inequality get smaller.

## Introduction

Does money buy happiness? This question has received extensive empirical attention since Richard Easterlin’s seminal work in 1974 (e.g. 1–3; see 4–5 for reviews). Easterlin (6) looked at results from 30 national surveys in 20 countries and concluded that “there is a clear indication that income and happiness are positively associated” (p. 99), and that “in every single survey, those in the highest status group were happier, on the average, than those in the lowest status group” (p. 100). Diener and Oishi (7) examined the income–happiness correlation in 40 countries and found that the mean income–happiness correlation was 0.13. According to Diener and Biswas-Diener (4), the income–happiness correlation in a national survey ranged from 0.12 to 0.18 in the USA, 0.06 to 0.15 in West Germany, and 0.17 to 0.27 in the Russian Federation. A recent meta-analysis of 335 studies found that the mean income–happiness correlation was 0.23 (8).

Inglehart and colleagues (9) analyzed data from 52 countries and found that the income–happiness correlation was stronger in poorer countries than in richer countries. That is, money seems to buy more happiness in poorer countries than in richer countries. In this light, it is interesting to note that the income–happiness correlation was 0.45 among 83 residents in the slums of Calcutta (10). The main idea is that among those who are struggling to meet their basic needs, more money means greater access to basic goods (e.g. drinking water, food, shelter). In contrast, it is believed that once the basic needs are met, more money does not necessarily help increase one’s happiness (9, 11).

Interestingly, recent research on money and happiness found that money seems to buy more happiness even among wealthy individuals whose basic needs are met. For instance, Killingsworth (12) analyzed the experience sampling data (i.e. momentary reports of happiness) from 33,391 employed, working-age adults living in the USA and found that self-reported happiness continues to increase as the participants’ household income increases, even beyond ${\$}$120,000. Moreover, Jebb and colleagues (13) analyzed the data from 164 countries and found that the income–happiness correlation was larger in *wealthier* countries rather than in poorer countries.

These recent findings suggest that the income–happiness correlation within a country (e.g. the USA, Argentina) might have changed over time. For instance, it is possible that in the USA, the income–happiness correlation was smaller in the 1970s and 1980s when the earlier data were being collected than in the 2010s and the 2020s when more recent data were collected. In contrast, it is possible that in some countries, the income–happiness correlation has become smaller over time, as their national economy grew. These divergent patterns could explain why the earlier cross-country studies (e.g. 9) found that the income–happiness correlation was larger among poorer than richer countries, whereas it was larger among richer rather than poorer countries in more recent cross-country studies (e.g. 13).

To our knowledge, none of the previous research has examined whether the income–happiness correlation has changed over time. This is a critical oversight because the importance of money changes over time in a given society (14, 15). Some countries might be becoming more materialistic, for example, while others might be becoming less materialistic over time (16).

Past research showed that the satisfaction of important life domains is more strongly associated with overall life satisfaction than the satisfaction of less important life domains (17). For instance, many people deem family relationships very important, whereas some do not. When the authors computed the correlation between family relationship satisfaction and life satisfaction, it was significantly stronger among those who deemed family relationships important than among those who did not. In the context of the present research, then, the income–happiness correlation should be larger as the importance of money increases, while it should be smaller as the importance of money decreases.

When does the importance of money increase or decrease? Based on Inglehart and colleagues’ findings (9), the end of materialism hypothesis predicts that the income–happiness correlation should get *smaller* as a society gets richer. This is because most people in a wealthy society are presumably no longer concerned about money per se, and instead are concerned about non-material issues such as self-expression. Under such a condition, self-expression should become a stronger predictor of happiness than money per se.

In contrast, there is an alternative possibility; as a society gets richer, people’s desires for material goods could also grow (18–21), and the importance of money could get even larger. For instance, some researchers found that American youths became more materialistic from the 1970s to the 1990s and their materialism remained high since then (15). In this scenario under the continuous materialism hypothesis, the income–happiness correlation should get larger as the economy grows.

Another important economic factor is the distribution of the national income. If the benefits of economic growth go disproportionately to the wealthy, then the poor feel that the world is unfair and report less happiness over time (e.g. 22). The larger the difference in happiness between the rich and the poor, the larger the correlation between income and happiness. In addition, growing income inequality is often driven by the rich getting richer (23), which in turn, provides more tendencies for ordinary citizens to engage in unfavorable, upward social comparisons (24). The frequency of upward social comparison is known to be detrimental to one’s happiness (25). In the current context, then, the economic gap between the rich and the poor should increase people’s tendency to engage in upward social comparison, which should in turn increase the happiness gap between the rich and the poor.

Moreover, income inequality is associated with more perceived competition (26), zero-sum thinking (27), and status anxiety (28). If income inequality has increased over time in a given country, then, the concern for money might have increased also. Indeed, previous research found that an increase in income inequality was associated with an increase in work hours (29). The increased concern for money, in turn, could translate into a larger income–happiness correlation (hereafter we call it the income inequality hypothesis). Furthermore, income inequality tends to be larger in countries where social welfare spending is low (e.g. Greece) than where social welfare spending is high (e.g. Sweden) because many welfare systems are funded through progressive taxation (30).

In a cross-sectional study of 29 countries, Ono and Lee (31) found that the income–happiness correlation was higher in countries where public social expenditure was lower than where it was higher. Ono and Lee’s findings are consistent with the hypothesis that the income–happiness correlation should be higher when income inequality is higher. As stated above, a shortcoming of the previous research is that it has not examined changes in the income–happiness correlation over time.

In sum, the main goals of the present research are to explore historical changes in the income–happiness correlation in diverse cultural, economic, and political contexts: the USA (Study 1), Japan (Study 2), Europe (Study 3), and Latin America (Study 4), and to test whether the historical changes in the income–happiness correlation are associated with GDP per capita and income inequality. The end of materialism hypothesis states that the income–happiness correlation will get smaller with an increase in GDP per capita. In contrast, the continuous materialism hypothesis states that the income–happiness correlation will get larger with an increase in GDP per capita. The income inequality hypothesis provides an alternative explanation for these conflicting hypotheses, instead predicting that the income–happiness correlation will get larger with an increase in income inequality.

### Study 1: The USA 1972 to 2018

The General Social Surveys (GSS) have included the 3-point happiness scale since its inception in 1972. We used this item with the self-reported household income from all the survey years. Because the value of the dollar has changed dramatically over time, the income categories in 1972 are not comparable to those in 2018. Furthermore, different years have different income categories (e.g. in 1972, there were 12 income categories; whereas in 1977 there were 20 categories). This inconsistency in response categories across years makes it questionable to use the income variable across different survey years in a single multilevel analysis. Thus, we used the meta-analytic framework, treating each year’s income–happiness correlation as an independent “study” result, as opposed to the multilevel framework in which the income variable has to be treated as equivalent across years. Finally, the original happiness scale was reversed to make the higher score indicate more happiness.

**Fig. 1.**
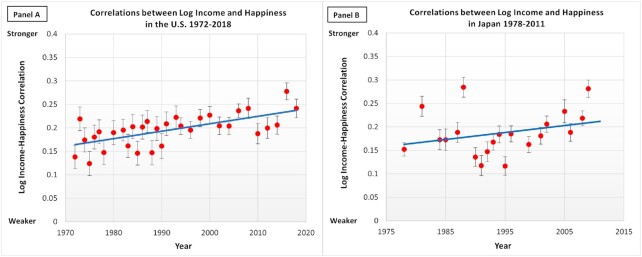
The log income–happiness correlation by survey year in the USA and in Japan. *Note:* The *x*-axis is the survey year in each country (begins at the first survey year available in each country: 1972 for the USA; 1978 for Japan). The *y*-axis is the correlation between the log of income and happiness (Fisher *z*-transformed). The error bar indicates the SE of the log income–happiness correlation. The blue line depicts the total trend (USA: *y* = 0.0016x–2.9762, *R*^2^ = 0.4224; Japan: *y* = 0.0015x–2.7704; *R*^2^ = 0.0817).

Figure 1 (left, panel A) shows the income–happiness correlation as a function of the survey year in the USA. The income–happiness correlation has steadily increased since the 1970s. Interestingly, however, in the post-Lehman shock surveys from 2010, 2012, and 2014, the correlations decreased. Fisher *z*-transformed income–happiness correlation coefficient was significantly associated with the survey year (*r*[30] = 0.65, *P* < 0.001) and the log-transformed GDP per capita (*r*[30] = 0.66, *P* < 0.001). Overall, the patterns of the results are generally consistent with the continuous materialism hypothesis, as the income–happiness correlation increased over the last 50 years during which GDP per capita has steadily increased.

**Fig. 2.**
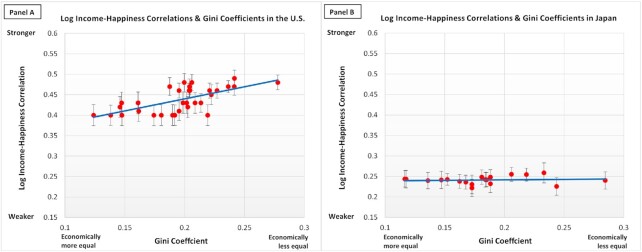
The log income–happiness correlation by the Gini coefficient in the USA and in Japan. *Note*. The *x*-axis indicates the Gini coefficient. The *y*-axis is the correlation between the log of income and happiness (Fisher *z*-transformed). The error bar indicates the SE of the log income–happiness correlation. The blue line depicts the total trend (USA: *y* = 0.5921x + 0.3215, *R*^2^ = 0.4281; Japan: *y* = 0.0232x + 0.237; *R*^2^ = 0.010).

Next, we tested the income inequality hypothesis. To the extent that the rich got richer, while the poor remained poor, the difference between the rich and the poor in the USA widened. The widening of the wealth gap might have translated into the widening of the happiness gap between the rich and the poor. As seen in Fig. 2, the Gini coefficients were highly positively correlated with the income–happiness correlation (*r*[30] = 0.66, *P* < 0.001). That is, in the years of greater income inequality, the correlation between income and happiness was stronger. The formal test with the classic meta-analysis function of the JASP program, using the restricted maximum likelihood estimation, showed that the household income inequality (Gini) significantly moderated the income–happiness correlation, *b* = 0.767 (SE = 0.149), *z* = 5.15, and *P* < 0.001. There was a substantial degree of heterogeneity in the income–happiness correlations between 1972 and 2018, *Q*(1, 31) = 73.20, and *P* < 0.001. Once we entered the Gini coefficient as the moderator, there was no longer heterogeneity in the remaining income–happiness correlations, *Q*(1, 30) = 38.35, *P* = 0.141.

#### Alternative specifications

Because the Gini coefficient is an omnibus index of inequality, it does not specify whether the above findings are driven mostly by the top 10% or the bottom 50%. We used the top 10% share and the bottom 50% share of the national income, respectively, as the moderator. The results were similar to the Gini findings. The income–happiness correlation was larger when the top 10% share of the national income was higher, *b* = 0.53 (SE = 0.11), *z* = 4.96, and *P* < 0.001. With the top 10% share of the national income included in the analysis, there was no longer heterogeneity in the remaining income–happiness correlations, *Q*(1, 30) = 39.75, *P* = 0.110. Conversely, the income–happiness correlation was smaller when the bottom 50% share of the national income was larger, *b* = −0.86 (SE = 0.18), *z* = −4.76, and *P* < 0.001. With the bottom 50% share of the national income included in the analysis, there was no longer significant heterogeneity, *Q*(1, 30) = 41.09, *P* = 0.085. Thus, using the alternative specifications, the income inequality hypothesis was supported in Study 1. In sum, Study 1 found support for the continuous materialism hypothesis and the income inequality hypothesis and did not find any support for the end of materialism hypothesis.

### Study 2: Japanese lifestyle surveys 1978 to 2011

Study 1 found patterns of the income–happiness correlation consistent with the continuous materialism hypothesis and the income inequality hypothesis, and inconsistent with the end of materialism hypothesis. As the economy grew, the income–happiness correlation increased rather than decreased over time. The USA has one of the most extreme forms of the winner-take-all economy and politics (32, 33). Thus, the USA could be an exception. Namely, it is possible that in other developed countries, the end of materialism hypothesis could still hold. Japan provides an ideal testing ground, as it is similar to the USA in terms of the GDP per capita, but different in many other dimensions. Most central to this research, the degree of income inequality is substantially smaller in Japan than in the USA (e.g. 34), and the Japanese are more aversive toward income inequality than Americans (35).

In order to test whether the increasing income–happiness correlation is specific to the USA, we next analyzed the Japanese Lifestyle Surveys, the nationally representative surveys conducted by the Japanese government from 1978 to 2011. The same 5-point scale life satisfaction question (“Are you satisfied or dissatisfied with your life in general?”) was included except in 2007, 2010, and 2011.

Figure 1 (right, panel B) shows the income–happiness correlation as a function of the survey year in Japan. Unlike in the USA, the income–life satisfaction correlation has not increased over time between 1978 and 2009 (*r*[18] = 0.29, *P* = 0.222). The Fischer-transformed income–life satisfaction correlation was not correlated with log-transformed GDP per capita (*r*[18] = 0.03, *P* = 0.895). Unlike Study 1, the income–life satisfaction correlation in Japan was not correlated with the Gini coefficient (*r*[17] = 0.10, *P* = 0.675), as well. Similarly, neither the top 10% share of the national income nor the bottom 50% share of the national income were associated with the size of the income–life satisfaction correlation: *r*(18) = 0.35, *P* = 0.131 for the top 10%; *r*(17) = −0.32, *P* = 0.182 for the bottom 50%. In sum, the patterns of the income–happiness correlations in Japan were not consistent with any hypotheses.

### Study 3: Eurobarometer surveys 1970 to 2002 and European social surveys 2004 to 2018

The USA and Japan showed different patterns of the income–happiness correlations over time. This could be due to the divergent pattern of economic growth in the USA and Japan: while the USA economy grew more or less linearly between 1972 and 2018 (Study 1), the Japanese economy stagnated between 1991 and 2011 (Study 2). In order to test the generalizability of our initial findings among Americans and Japanese, we examined the income–life satisfaction correlations over time among 16 European countries. We analyzed the Eurobarometer surveys and the European Social Surveys (ESS). The Eurobarometer included the 4-point scale life satisfaction question in most years, whereas the ESS included the 10-point scale life satisfaction question in most years. We chose the countries included both in the Eurobarometer and ESS in the following analyses. (Eurobarometer and ESS contain data from countries have changed their names/boundaries historically [i.e. Great Britain and West-Germany in Eurobarometer; the UK and Germany in ESS]. For consistency, we used “the UK” and “Germany” across the studies.) This resulted in 16 countries with a total income–life satisfaction correlation of 414. Many of the recent Gini coefficients were not available (for instance, the World Bank’s database [https://data.worldbank.org/indicator] does not have any Gini coefficients for France before 1978; furthermore, years 1979 to 1988 are missing; in contrast, the World Inequality database [https://wid.world/] has the top 10% share and the bottom 50% share of the national income every year since 1915 for France), whereas the top 10% share and the bottom 50% share were consistently available (https://wid.world/). Thus, in Studies 3 and 4, we used these two indices of income inequality as opposed to the Gini coefficient.

**Fig. 3.**
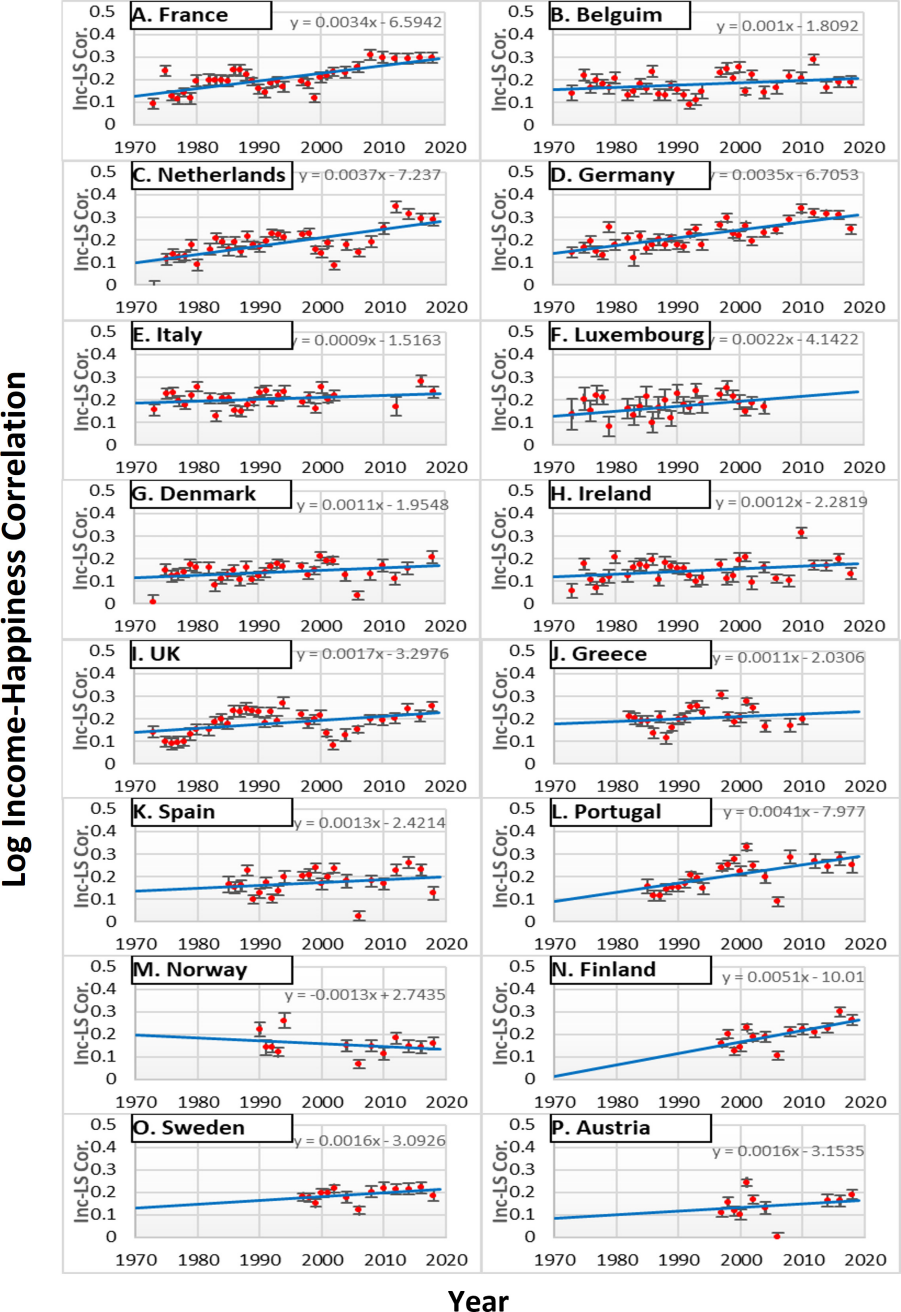
The log income–happiness correlation by survey year by Country in Europe (1970 to 2018). *Note*. Inc-LS Cor. Indicates the correlations between log income and life satisfaction (Fisher *z*-transformed). The error bar indicates the SE of the correlation coefficient.

Like in Studies 1 and 2, we first computed an income–life satisfaction correlation for each year for each country (e.g. *r* = 0.13 in 1976, *r* = 0.11 in 1977, *r* = 0.14 in 1978 etc. in France; *r* = 0.17 in 1976, *r* = 0.20 in 1977, *r* = 0.18 in 1978 etc. in Belgium). We then computed a correlation between income–life satisfaction correlations (Fisher *z*-transformed) and the survey year, GDP per capita (log-transformed), the top 10%, and the bottom 50% share in each of the 16 countries, respectively. Table 1 shows these correlations. Figure 3 shows the income–happiness correlation as a function of the survey year across countries in Europe.

**Table 1.** The correlations between the log income–happiness correlations and the survey year (yearly trend), GDPpc (LN), and two-income inequality indices (top10% share of the national income and bottom 50% share of the national income).

Country	Yearly Trend	GDPpc (LN)	Top 10% share	Bottom 50% share
GSS (1972 to 2018)				
USA	.65**	.66**	.63**	−0.62**
Japanese Lifestyle Surveys (1978 to 2011)				
Japan	.29	.03	.35	−0.32
Eurobarometer ± European Social Surveys (1970 to 2018)				
France	.74**	.65**	.13	−0.11
Belgium	.29	.30	.04	.12
Netherlands	.69**	.48*	.37	−0.61**
Germany	.78**	.72**	.79**	−0.79**
Italy	.28	.20	.33	−0.29
Luxembourg	.31	.33	.43	−0.40
Denmark	.30	.26	−0.07	−0.17
Ireland	.32	.25	.16	.02
UK	.42*	.38*	.43*	.12
Greece	.21	.01	.44*	−0.42
Spain	.24	.11	−0.35	.28
Portugal	.63*	.67**	.57*	−0.43*
Norway	−0.28	−0.36	−0.36	.33
Finland	.68*	.38	.02	−0.59*
Sweden	.40	.33	−0.71*	−0.11
Austria	.20	.07	.69*	−0.70*
Meta-analytic *r* [95% CI]	.44 [.29; 0.57]	.35 [.20; 0.49]	.22 [−0.03; 0.44]	−0.26 [−0.45; −0.05]
Latinobarometro(1997 to 2018)				
Argentina	−0.32	−0.50	.44	−0.48
Bolivia	−0.39	−0.07	.05	−0.11
Brazil	−0.49*	−0.37	−0.42	.0005
Chile	−0.21	−0.23	.34	−0.66*
Colombia	−0.36	−0.12	.43	−0.46
Costa Rica	.21	.03	−0.06	.06
Dominican	−0.38	−0.33	.33	−0.35
Ecuador	.42	.30	−0.21	.15
El Salvador	−0.56*	−0.47	.18	−0.47
Guatemala	−0.18	.20	−0.37	.32
Honduras	.0002	.14	−0.04	.10
Mexico	.03	.33	.24	−0.31
Nicaragua	−0.05	−0.09	.28	−0.22
Panama	.01	−0.02	.18	−0.09
Paraguay	−0.09	.06	.25	−0.17
Peru	−0.01	.36	−0.32	.40
Uruguay	−0.11	.05	.06	.06
Venezuela	−0.18	−0.003	.32	−0.40
Meta-analytic *r* [95% CI]	−0.15 [−0.28; −0.02]	−0.04 [−0.18; 0.10]	.09 [−0.05; 0.23]	−0.16 [−0.31; 0.003]

*Note*. *P < 0.05. **P < 0.01. The correlation coefficients between the within-country log income–happiness correlations (Fisher *z*-transformed) and the survey year, GDP per capita (Log-transformed), top 10% share of the national income, and bottom 50% share of the national income of the year.

The first column of Table 1 shows the correlation between the income–life satisfaction correlation and the year of the survey. A positive correlation (e.g. *r* = 0.78 in Germany, 0.74 in France) indicates that the income–life satisfaction correlation increased over time in that country, whereas, a negative correlation (e.g. *r* = −0.28 in Norway) indicates that the income–life satisfaction correlation decreased over time in that country. Like in the USA, the income–life satisfaction correlation has substantially increased between 1970 and 2018 in France, Germany, Finland, Portugal, the UK, and the Netherlands (*r*s ranged from 0.42 to 0.78). In contrast, the income–life satisfaction correlation has decreased in Norway. We meta-analyzed the correlations between the within-country income–life satisfaction correlation (Fisher *z*-transformed) and the year of the survey, with the random-effects model, using the *meta* package in R. The meta-analytic mean correlation of yearly trends was 0.44 (95% CI: 0.29; 0.57; *t* = 5.80, *P* < 0.001) among 16 European countries. That is, on average, the income–happiness correlation increased over time among 16 European countries.

The second column of Table 1 shows the correlation between the income–life satisfaction correlation and GDP per capita (log-transformed). A positive correlation (e.g. *r* = 0.72 in Germany, *r* = 0.67 in Portugal) indicates that the income–life satisfaction correlation was larger when GDP per capita was higher, whereas a negative correlation (e.g. *r* = −0.36 in Norway) indicates that the income–life satisfaction correlation was smaller when GDP per capita was lower. Our results showed that the size of an income–life satisfaction correlation was highly positively correlated with GDP per capita (log-transformed) in Portugal, France, Germany, the Netherlands, and the UK (*r*s ranged from 0.38 to 0.72). The meta-analytic mean correlation of the income–life satisfaction correlation with GDP per capita was 0.35 (95% CI: 0.20; 0.49; *t* = 4.81, *P* < 0.001) among 16 European countries. These findings are consistent with the continuous materialism hypothesis, and inconsistent with the end of materialism hypothesis.

Next, we examined whether the income–life satisfaction correlations were larger in the year of larger income inequality. The third column of Table 1 shows the correlation between the income–life satisfaction correlation and the top 10% share of the national income. A positive correlation (e.g. *r* = 0.79 in Germany) indicates that the income–life satisfaction correlation got larger, as the top 10% share grew, whereas a negative correlation (e.g. *r* = −0.71 in Sweden) indicates that the income–life satisfaction correlation got smaller, as the top 10% share grew. Like in the USA, the income–life satisfaction correlations were substantially higher in the year of a higher share of the national income being held by the richest 10% of the population in Germany, Portugal, Austria, Greece, and the UK. In contrast, it was smaller in the year with a higher top 10% share in Sweden. Overall, the meta-analytic mean correlation of the income–life satisfaction correlation with the top 10% share was 0.22 (95% CI: −0.03 0.44; *t* = 1.85, *P* = 0.083). That is, across 16 European countries, on average, the income–life satisfaction correlation tended to be larger in the year of the higher concentration of the national wealth in the richest 10% of the population.

The last column of Table 1 indicates the correlation between the income–life satisfaction correlation and the bottom 50% share of the national income. A negative correlation (e.g. *r* = −0.79 in Germany) in this column means that the income–life satisfaction correlation was smaller in the year of the larger bottom 50% share of the national income. Like in the USA, the income inequality hypothesis was supported in Germany, Finland, the Netherlands, Portugal, and Austria. In contrast, it was not supported in Belgium, the UK, Ireland, Spain, and Norway. Overall, the meta-analytic mean correlation of the income–life satisfaction correlation with the bottom 50% share was −0.26 (95% CI: −0.45; −0.05; *t* = −2.65, *P* = 0.018). Thus, on average, the income–life satisfaction correlations were larger when the bottom 50% share was smaller, consistent with the income inequality hypothesis.

In sum, the meta-analyses showed that the income–life satisfaction correlations between 1970 and 2018 among 16 European countries on average were similar to those in the USA. Like in the USA, the income–life satisfaction correlations increased over time. The income–life satisfaction correlation was higher in the year of higher GDP per capita. It was marginally higher in the year of higher top10% share, whereas significantly lower in the year of higher bottom 50% share of the national income. The European data on average provided support for the continuous materialism hypothesis and the income inequality hypothesis, and no support for the end of materialism hypothesis.

### Study 4: Latinobarometro 1997 to 2018

Studies 1 to 3 showed that the income–life satisfaction correlation (a) increased over time in the USA and European countries on average, (b) coincided with an increase in GDP per capita, and (c) an increase in income inequality in the USA and European countries on average. We explored whether the patterns of the income-happiness/life satisfaction correlations found in the USA and European countries are generalizable among Latin American countries. In the USA and several European countries, income inequality has increased as the economy grew over the last 40 years. Latin America is an interesting case in that, unlike in the WEIRD (Western Educated Industrialized Rich Democratic) countries, income inequality has decreased over the last 40 years as the economy grew (36, 37). We tested the trends of the income–life satisfaction correlation over time among 18 Latin American countries over the last 2 decades.

We analyzed the Latinobarometro data from 1997 to 2018. Life satisfaction was assessed with the 4-point scale (1 = not very satisfied to 4 = very satisfied). Income was not directly assessed. However, respondents’ assessment of their income was assessed every year using the 4-point scale (“Does your salary and the total of your family’s salary allow you to satisfactorily cover your needs?” 1 = Does not cover, there are great difficulties to 4 = covers them well, I can save”). We used these two items to calculate the correlation between income and life satisfaction each year for each country.

**Fig. 4.**
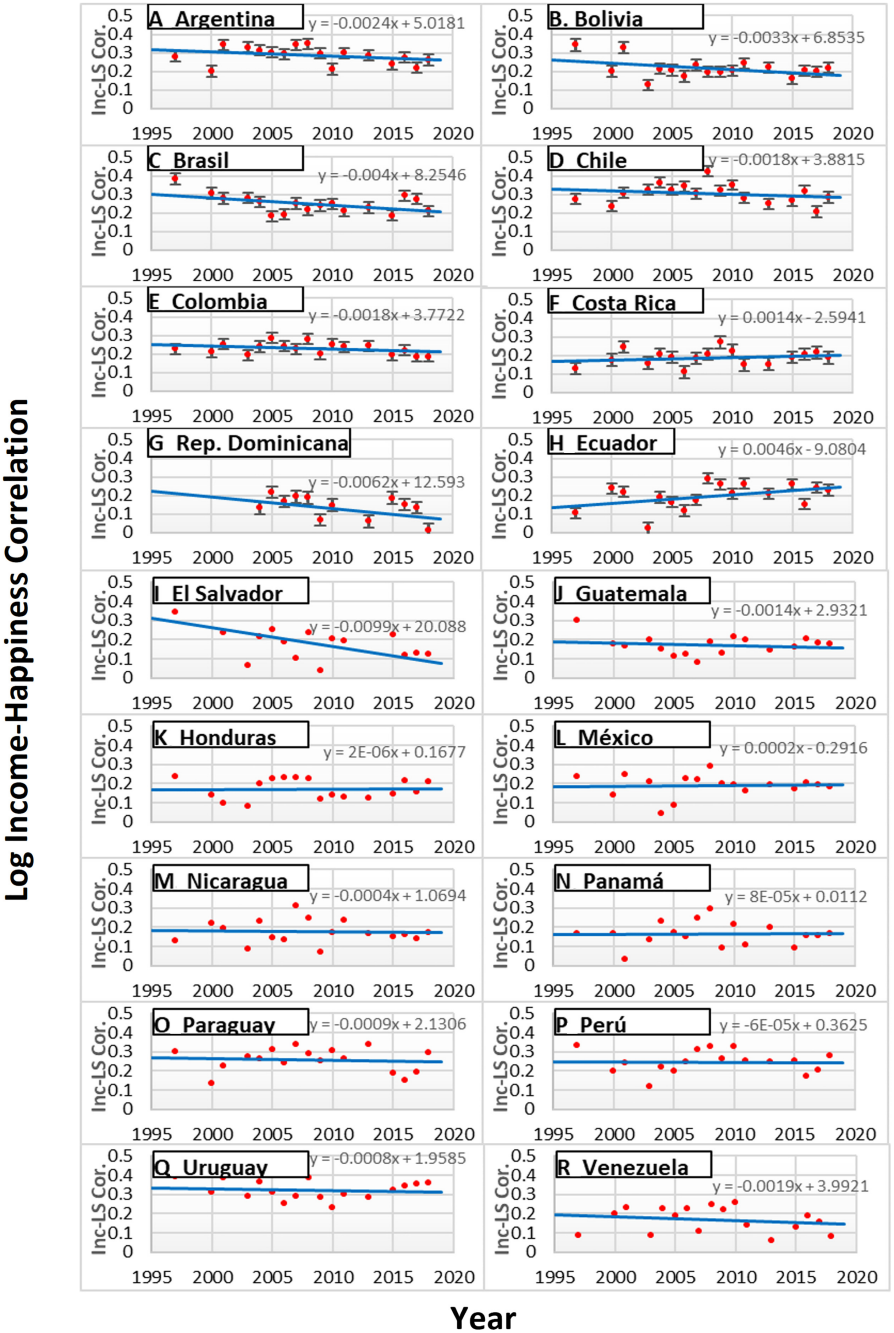
The log income–happiness correlation by survey year by country in Latin America (1997 to 2018). *Note*. Inc-LS Cor. Indicates the correlations between log income and life satisfaction (Fisher *z*-transformed). The error bar indicates the SE of the correlation coefficient.

Like in Studies 1 to 3, the income–life satisfaction correlation was computed for each year for each country, separately. First, whereas the income–life satisfaction correlations have grown larger over time in the USA and several European countries, on average, the income–life satisfaction correlation has become *smaller* among Latin American countries (see Fig. 4), as indicated by many negative correlations in the first column of Table 1: the meta-analytic *r* = −0.15 (95% CI: −0.28; −0.02; *t* = −2.43, *P* = 0.027).

Furthermore, the second column of Table 1 shows that, unlike the USA and several European countries, the income–life satisfaction correlation was not associated with GDP per capita among 18 Latin American countries: meta-analytic *r* = −0.04 (95% CI: −0.18; 0.10, *t* = −0.63, *P* = 0.53). Finally, the patterns of the income–life satisfaction correlation with income inequality indices (the 3rd and 4th columns of Table 1) were similar to those found in the USA and Europe. The income–life satisfaction correlation was smaller when the bottom 50% share was larger: meta-analytic *r* = −0.16 (95% CI: −0.31; 0.003; *t* = −2.07, *P* = 0.054), although the correlation with the top 10% share of income was not statistically significant (meta-analytic *r* = 0.09 [95% CI: −0.05; 0.23; *t* = 1.38, *P* = 0.186]). Thus, Latin American data were mostly consistent with the income inequality hypothesis.

### The multilevel meta-analyses of studies 1 to 4

We next meta-analyzed the income-happiness/life satisfaction correlations from the USA, Japan, 16 European countries, and 18 Latin American countries simultaneously to get an overall estimate of the effect size across 36 countries. There were 768 income-happiness/life satisfaction correlations from 36 countries. We meta-analyzed them with the multilevel model, using the *metafor* package in *R* (Level 1 = within-country; Level 2 = between-country). The base model in which the income–happiness correlation was predicted by year only showed that the intercept was 0.173 (*SE* = 0.009), *t* (766) = 19.85, *P* < 0.001. On average, the *z*-transformed income–happiness correlation was 0.173 in the first year of the survey across 36 countries, which were slightly higher than Diener and Oishi’s (7) earlier review (*r* = 0.13, which did not include Latin America) and lower than the most recent meta-analysis: *r* = 0.23 (8). The slope for the year was 0.001 (*SE* = 0.0014), *t* = 7.50, *P* < 0.001, meaning that one year corresponded to an increase of 0.001 in the income–happiness correlation on average. The results from 3 different sets of meta-analyses are shown in Table 2. Model 1 included year, three dummies (dummy 1 = Japan; dummy 2 = Europe, 3 = Latin America), and interaction terms (region dummies with the yearly trend). The results from Model 1 showed that (a) the income–happiness correlations in the USA were on average higher than Japan, Europe, and Latin America, (b) that there was no difference between Japan and the USA, nor a difference between Europe and the USA in the yearly trend, but (c) that the yearly trend of Latin American countries was significantly different from that of the USA.

**Table 2.** Multilevel meta-analyses of studies 1 to 4.

	*b*	*SE*	*t*	*df*	*p*	CILL	CIUL	*Q*	*df*	*P*
Base Model								5349.68	766	<0.001
**Intercept**	**.173**	**.009**	**19.85**	**766**	**<0.001**	**.1560**	**.1903**			
**Year**	**.001**	**.0002**	**7.50**	**766**	**<0.001**	**.0011**	**.0018**			
Model 1								4721.06	760	<0.001
**Intercept**	**.164**	**.045**	**3.65**	**760**	**<0.001**	**.0759**	**.2526**			
**Year**	**.002**	**.001**	**2.50**	**760**	**.013**	**.0003**	**.0029**			
Japan	−0.002	.066	−0.04	760	.972	−0.1312	.1266			
Euro	−0.031	.047	−0.67	760	.505	−0.1226	.0604			
Latin	.066	.047	1.43	760	.154	−0.0250	.1579			
Japan*year	−0.0001	.001	−0.06	760	.951	−0.0028	.0026			
Euro*year	.001	.001	.67	760	.503	−0.0009	.0018			
Latin*year	−**0.003**	**.001**	−**3.75**	**760**	**<0.001**	−**0.0047**	−**0.0015**			
Model 2								4593.74	719	<0.001
**Intercept**	**.197**	**.042**	**4.65**	**719**	**<0.001**	**.1139**	**.2805**			
Japan	−0.011	.060	−0.18	719	.856	−0.1292	.1073			
Euro	−0.018	.044	−0.42	719	.675	−0.1042	.0675			
Latin	.015	.044	.35	719	.727	−0.0706	.1011			
**GDPpc**	**.009**	**.002**	**3.79**	**719**	**<0.001**	**.0042**	**.0133**			
**Top10%**	**.009**	**.002**	**4.33**	**719**	**<0.001**	**.0050**	**.0133**			
GDPpc*Top10%	.003	.003	1.19	719	.236	−0.0021	.0083			
Model 3								4342.94	695	<0.001
**Intercept**	**.195**	**.042**	**4.64**	**695**	**<0.001**	**.1123**	**.2769**			
Japan	−0.008	.060	−0.14	695	.892	−0.1250	.1088			
Euro	−0.014	.043	−0.33	695	.743	−0.0989	.0706			
Latin	.019	.043	.45	695	.655	−0.0655	.1042			
**GDPpc**	**.008**	**.002**	**3.43**	**695**	**<0.001**	**.0035**	**.0128**			
**Bottom50%**	−**0.010**	**.002**	−**4.93**	**695**	**<0.001**	−**0.0146**	−**0.0063**			
GDPpc*Bottom50%	−**0.006**	**.003**	−**2.14**	**695**	**.033**	−**0.0111**	−**0.0005**			

*Note*. Bold-faced lines indicate statistically significant findings. Asterisks indicate interaction terms. The z-transformed log income–happiness correlation was predicted by yearly trend (the first year of the survey coded as 0), Japan (Japan = 1; the rest = 0), Euro (Euro = 1; the rest = 0), Latin America (Latin American = 1; the rest = 0), and three interaction terms in Model 1. The *z*-transformed log income–happiness correlation was predicted by Japan (Japan = 1; the rest = 0), Euro (Euro = 1; the rest = 0), Latin America (Latin American = 1; the rest = 0), GDP per capita (log-transformed, then *z*-scored within each country), top 10% (z-scored within each country), and the interaction term in Model 2. The *z*-transformed log income–happiness correlation was predicted by Japan (Japan = 1; the rest = 0), Euro (Euro = 1; the rest = 0), Latin America (Latin American = 1; the rest = 0), GDP per capita (log-transformed, then *z*-scored within each country), bottom 50% (*z*-scored within each country), and the interaction term in Model 3. CILL = Confidence Interval Lower Limit. CIUL = Confidence Interval Upper Limit. Q denotes Q statistics, the indicator of heterogeneity of effect sizes.

Model 2 tested whether the income–happiness correlations were larger in the years of higher levels of GDP per capita and the top 10% of share simultaneously (Model 2). Controlling for the regional differences in the mean income–happiness correlation and top 10% share, one standard deviation increase in GDP per capita (e.g. 1 SD of log GDP was 0.22 for the USA) corresponded to a 0.009 increase in the income–happiness correlation, *t* = 3.79, *P* < 0.001. Thus, across 36 countries, on average, the income–happiness correlation was larger when GDP per capita was higher, supporting the continuous materialism hypothesis as opposed to the end of materialism hypothesis. In addition, this analysis showed that one standard deviation increase in the top 10% share of the national income was, on average, associated with a 0.009 increase in the income–happiness correlation, *t* = 4.33, *P* < 0.001, supporting the income inequality hypothesis (for the time trends in GDP and the top 10% share across countries, see Figures S1 to S8, Supplementary Material 2).

Model 3 tested whether the income–happiness correlation was larger in the years of a smaller share of the bottom 50% across the 36 countries. The results from Model 3 showed that controlling for the regional differences and the bottom 50% share, the income–happiness correlation was larger in the year of higher GDP per capita, again supporting the continuous materialism hypothesis. One standard deviation increase in GDP per capita was associated with a 0.008 *increase* in the income–happiness correlation. Consistent with the income inequality hypothesis, the income–happiness correlation was smaller in the year of a larger bottom 50% share of the national income (i.e. less income inequality) than in the year of a smaller bottom 50% share of the national income (i.e. more income inequality). One standard deviation increase in the bottom 50% share of the national income was associated with a 0.010 *decrease* in the income–happiness correlation. Finally, there was a significant interaction between GDP per capita and the bottom 50% share, such that the income–happiness correlations were larger in the year of higher GDP per capita and the smaller bottom 50% share (i.e. more income inequality).

### General discussion

We started our investigation to explore historical changes in the income–happiness correlation in diverse samples. In the USA the income–happiness correlation has *increased* substantially since 1972 (Fig. 1). In contrast, the income–life satisfaction correlation did not increase from 1978 to 2011 in Japan (Study 2). Study 3 analyzed the Eurobarometer and European Social Surveys from 1970 to 2019. The meta-analysis largely replicated the findings from the USA On average, the income–life satisfaction correlation among the 16 European countries *increased* over time. Finally, we examined the patterns of the income–life satisfaction correlation among Latin Americans from 1997 to 2018. Unlike in the USA, Japan, and Europe, the income–life satisfaction correlation *decreased* over time in Latin America.

Overall, the historical patterns of the income–happiness correlation observed in the USA and several European countries, most notably Germany, the UK, the Netherlands, and Portugal, were consistent with the continuous materialism hypothesis and the income inequality hypothesis. In contrast, the patterns observed in Latin America were only consistent with the income inequality hypothesis.

In support of the continuous materialism hypothesis, the income–happiness correlation became larger as GDP per capita increased in the USA, France, Germany, UK, the Netherlands, and Portugal. In these countries, when the national economy was stronger, money appeared to be more important to people’s happiness than when the national economy was weaker. These findings are consistent with Robert Frank’s (1999) idea of luxury fever at a time of the economic boom. Even when survival per se is no longer an issue, money still matters to many people. Money can buy them a better car, a smartphone, and a bigger house. As people’s financial situations improve, the material possessions deemed necessary increase, as well (38–40). As desires increase, money continues to matter even more, making the income–happiness correlation stronger, not weaker.

Furthermore, in the USA and European countries on average, when income inequality was larger, money appeared to be more important than when income inequality was smaller. These findings are consistent with the previous findings on the psychology of inequality: namely, inequality appears to amplify people’s tendencies to perceive more competition in society (26), believe in the zero-sum nature of society (27), and engage in unfavorable upward social comparison (24), in particular among the poor. Likewise, fairness and general trust might be eroded particularly among the poor at a time of high levels of income inequality (22). This could, in turn, increase the happiness gap between the rich and the poor, inflating the income–happiness correlation. In addition, Laurin et al. (38) findings on fairness and self-regulation suggest that the poor might be more likely to give up trying to achieve long-term goals at a time of vast inequality than small inequality. This in turn could decrease the happiness of the poor, resulting in a larger happiness gap between the rich and the poor. In the future, it is important to directly measure changes in desires and the importance of money, as well as these psychological mechanisms in the context of changes in the income–happiness correlation.

Whereas the continuous materialism hypothesis and the inequality hypothesis were equally associated with historical changes in the income happiness correlations in the USA and Europe, the income inequality hypothesis was the only hypothesis that received empirical support in Latin America. Since 1997 (the first year of our data in Latin America), income inequality has declined in most Latin American countries. For instance, in Argentina, the bottom 50% share of the national income increased from 10.6% in 2000 to 17.8% in 2018. In contrast, during the same period, the bottom 50% share went down from 15.1% to 13.5% in the USA. Thus, in many Latin American countries, the decreased levels of income inequality might have reduced perceived competition, zero-sum thinking, and unfavorable upward social comparison, reducing the happiness gap between the rich and the poor. Again, these psychological mechanisms should be explicitly tested in the context of Latin America.

Although we analyzed most of the nationally representative data available today, the years covered by these surveys were still limited. For instance, Study 2 included only 20 surveys, limiting the statistical power to detect only large effect sizes. Second, it is interesting that among European countries, several countries such as the Netherlands, Germany, UK, and Portugal were similar to the USA (a strong positive correlation between GDPpc and income inequality); The yearly trend in Norway was quite different from other European countries. It is important to identify a moderator to explain these cross-national variations in the future. It is also important to examine alternative explanations to the continuous materialism (e.g. inflation, unemployment rate) and income inequality hypotheses (e.g. social welfare spending).

Finally, although it is unlikely that a change in the income–happiness correlation causally affects a change in income inequality or GDPpc, our design does not allow for causal inference. As more data become available, a more stringent time series analysis (e.g. Granger’s causality test) should be conducted to discern a causal relation better.

### Conclusion

We found that the income–happiness correlation has increased in the USA and several European countries (e.g. Germany, the Netherlands, Portugal) since 1972 as GDP per capita and income inequality increased. In contrast, in Latin American countries on average, the income–happiness correlation has decreased since 1997. In Japan and several other countries (e.g. Denmark, Panama), the income–happiness correlation has not significantly changed. These divergent historical trends of the income–happiness correlation were associated with economic conditions. The income–happiness correlation tends to get larger as GDP per capita increases, providing support for the continuous materialism hypothesis. Independently, the income–happiness correlation tends to get larger as top 10% share of the national income increases and bottom 50% share of income decreases, providing support for the income inequality hypothesis. These findings suggest that on average, money becomes more central to one’s happiness under high GDP per capita and high income inequality. That is, in developed countries such as the USA, Germany, and the Netherlands, materialism is persistent. In understanding the role of money in happiness, researchers need to account for not just national wealth but also how national wealth is distributed across social class, as both national wealth and inequality appear to be associated with materialism.

## Materials and methods

All data, the analytic codes, and supplementary materials are available in the Open Science Framework at https://osf.io/x5gcw. For the descriptive statistics and correlations between variables, see Supplementary Material 1.

### Study 1 method

#### General social surveys

Participants were 64,814 Americans (28,614 men, 36,200 women) age 18 and over. Out of 64,814 respondents, 52,033 were white, 9,187 were black (3,594 were “Other”). The mean age was 46.10 (SD = 17.54). The mean number of respondents who answered the happiness item per survey year was 1,692.91 (SD = 481.13), ranging from 1,173 to 2,627.

The 3-point scale happiness item (“Taken all together, how would you say things are these days—would you say that you are very happy, pretty happy, or not too happy?”) was included in every survey. The responses were reverse scored such that 1 = not too happy, 2 = pretty happy, and 3 = very happy.

Total family income was measured by different items across different survey years: a 12-category item: “1 = less than ${\$}$2,000, 2 = ${\$}$2,000 to ${\$}$3,999. . .to 12 = ${\$}$30,000+” in 1972, by a 12 category item: “1 = less than ${\$}$1,000, 2 = ${\$}$1,000 to ${\$}$2,999. . .12 = ${\$}$25,000 or more” in 1973, 1974, 1975, and 1976, by a 16 category item: “1 = less than ${\$}$1,000, 2 = ${\$}$1,000 to 2,999. . .12 = ${\$}$17,500 to 19,999. . .16 = ${\$}$50,000+” in 1977, 1978, 1980, by a 17 category item “1 = less than ${\$}$1,000, 2 = ${\$}$1,000 to 2,999. . .17 = ${\$}$50,000 + in 1982, 1983, 1984, and 1985, by a 20 category item “1 = less than ${\$}$1,000, 2 = ${\$}$1,000 to 2,999. . .20 = ${\$}$60,000 or more” in 1986, 1987, 1988, 1989, and 1990, and by a 21 category item: “1 = less than ${\$}$1,000, 2 = ${\$}$1,000 to 2,999. . .21 = ${\$}$75,000 or more” in 1991, 1993, 1994, 1996, by a 23 category item: “1 = less than ${\$}$1,000, 2 = ${\$}$1,000 to 2,999. . .23 = ${\$}$110,000 or more” in 1998, 2000, 2002, 2004, and by a 25 category item “1 = less than ${\$}$1,000, 2 = ${\$}$1,000 to 2,999. . .25 = ${\$}$150,000+” in 2006, 2008, 2010, 2012, 2014, and by a 26 category item: “1 = less than ${\$}$1,000, 2 = ${\$}$1,000 to 2,999. . .26 = ${\$}$170,000+” in 2016 and 2018. We then converted each category of the total family income in each survey to the dollar using the midpoint value (e.g. 1 = < ${\$}$2,000 → ${\$}$1,000, 2 = 2,000 to 3,999 → ${\$}$3,000), while the highest value was multiplied by 1.5 (e.g. 12 = ${\$}$25,000 or more → ${\$}$37,500). Finally, we log-transformed them. All the income–happiness correlations reported in the text were based on the log-transformed midpoint values. For the category values, midpoint values, historical exchange rates, and log-transformed values in Study 1 and the other studies, see Supplementary Material 3 (https://osf.io/x5gcw)

GDP per capita was taken from https://wid.world/. Gini coefficient was taken from Table H-4 in https://www.census.gov/data/tables/time-series/demo/income-poverty/historical-income-households.html.

### Study 2 method

Participants were 80,857 Japanese (37,212 men; 40,254 women) age 15 to 88. The mean age was 44.54 (SD = 15.57). The mean number of respondents for the life satisfaction question used per year was 2,846.61 (SD = 1,548.23), ranging from 1,692 to 7,405.

The 5-point scale life satisfaction item (1 = dissatisfied, 2 = rather dissatisfied, 3 = neither satisfied nor dissatisfied, 4 = rather satisfied; 5 = satisfied) was included in 1978, 1981, 1984, 1985, 1987, 1988, 1990, 1991, 1992, 1993, 1994, 1995, 1996, 1999, 2001, 2002, 2005, 2006, 2008, and 2009.

Income was measured differently in different surveys. In 1978, 1981, 1984, 1987, 1988, 1990, it was measured by a 21-point scale (1 = less than 750,000 yen; 2 = 750,000 to 1.25 mil yen; 3 = 1.25 to 1.75 mil yen. . .21 = over 10.25 mil yen). In 1985, it was measured by a 27-point scale (1 = None, 2 = less than 500,000 yen; 3 = 500,000 to 1 mil yen; 4 = 1 to 1.5 mil yen. . .27 = over 20 mil yen). In 1991, it was measured by a 23-point scale (1 = less than 1.25 mil yen; 2 = 1.25 to 1.75 mil yen; 3 = 1.75 to 2.25 mil yen, 4 = 2.25 to 2.75 mil yen. . .23 = over 30mil yen). In 1992, 1996, 1999, 2002, 2005, 2008, and 2011, it was measured by an 8-point scale (1 = less than 2mil yen; 2 = 2 to 4 mil yen; 3 = 4 to 6 mil yen; 4 = 6 to 8 mil yen; 5 = 8 to 10 mil yen; 6 = 10 to 12 mil yen; 7 = 12 to 14 mil yen, 8 = over 14 mil yen). In 1993, it was measured by a 12-point scale (1 = less than 750,000 yen; 2 = 750,000 to 2.25 mil yen; 3 = 2.25 to 3.75 mil yen. . .12 = over 15.75 mil yen). In 1994, it was measured by a 13-point scale (1 = less than 500,000 yen; 2 = 500,000 to 1 mil yen; 3 = 1 to 1.5 mil yen; 4 = 1.5 to 2 mil yen. . .13 = over 10 mil yen). In 1995, it was measured by a 9-point scale (1 = less than 1 mil yen; 2 = 1 to 2 mil yen; 3 = 2 to 4 mil yen; 4 = 4 to 6 mil yen. . .9 = over 14 mil yen). In 2001, it was measured by a 10-point scale (1 = less than 20mil yen; 2 = 2 to 4 mil yen; 3 = 4 to 6 mil yen; 4 = 6 to 8 mil yen. . .10 = over 20 mil yen). In 2006 and 2007, it was measured by a 10-point scale (1 = less than 1mil yen; 2 = 1 to 3 mil yen; 3 = 3 to 5 mil yen; 4 = 5 to 7 mil yen. . .10 = over 20 mil yen). In 2009 and 2010, it was measured by a 5-point scale (1 = less than 1 mil yen; 2 = 1 to 3 mil yen; 3 = 3 to 5 mil yen; 4 = 5 to 10 mil yen; 5 = over 10 mil yen). The income variables were converted to yen, then log-transformed, as in Study 1.

GDP per capita was taken from https://wid.world/. Gini coefficient was taken from https://data.worldbank.org/indicator.

### Study 3 method

Study 3 combined annual datasets from Eurobarometer Surveys 1973 to 2002 and biennial datasets from European Social Surveys 2004 to 2018. Participants from Eurobarometer Surveys were 659,140 Europeans (*M*_age_ = 43.16, *SD*_age_ = 17.92, 51.7% women). Participants from European Social Surveys were 422,985 Europeans (*M*_age_ = 48.29, *SD*_age_ = 18.63, 53.8% women). The mean number of respondents who answered the life satisfaction item per survey year was 28,885.73 (*SD* = 14,422.27), ranging from 8,767 to 54,151.

As for the life satisfaction item, every survey year of Eurobarometer Surveys included the 4-point scale life satisfaction item (“On the whole, are you very satisfied, fairly satisfied, not very satisfied, or not at all satisfied with the life you lead?”), except for 1974 and 1996. Every survey year of European Social Surveys included the life satisfaction item (“How satisfied with life as a whole”), with different answer scales such as 10-points (0 = extremely dissatisfied; 10 = extremely satisfied). As for the household income item, both Eurobarometer Surveys and European Social Surveys used different categories and scales across countries and years. There are the variables named “income” in Eurobarometer Surveys and “hinctnt” and “hinctnta” in European Social Surveys, which used different categories and scales in each of the 16 countries. Compared to the other survey years that used money values, the 2008 to 2018 surveys used income deciles in each nation. All the income variables were converted to the local currency and log-transformed, as in Studies 1 and 2.

GDP per capita, the top 10% share of the national income, the bottom 50% share of the national income were taken from https://wid.world/.

### Study 4 method

Data across 18 Latin American countries were derived from Latinobarometro 1997 to 2018 (https://www.latinobarometro.org/). The total number of participants who answered life satisfaction and income item were 431,148 (*M*_age_ = 40.05, *SD*_age_ = 16.47, 51.5% women). Ethnicity/race data are available only in 2007 to 2018 (0.66% Asian, 5.45% Black, 9.33% Indigenous, 46.83% Mestizo, 5.95% Mulato, 29.86% White, 1.94% Other race). The mean number of respondents who answered the life satisfaction item per survey year was 20,873 (*SD* = 1560.91), ranging from 17,601 to 22,615.

Life satisfaction was measured with the 4-point scale (“Generally speaking, would you say you are satisfied with your life? Would you say you are. . .? 1 = not very satisfied to 4 = very satisfied).

Income was assessed using the 4-point scale (“Does your salary and the total of your family’s salary allow you to satisfactorily cover your needs? 1 = Doesn’t cover, there are great difficulties to 4 = covers them well, I can save”).

Skewness and kurtosis were within the acceptable range (skewness < 2.00; kurtosis < 7.00, according to 39) as follows: 1997 (0.17; −0.56), 1998 (0.12; −0.65), 2000 (0.09; −0.58), 2001 (0.11; −0.73), 2002 (0.01; −0.72), 2003 (−0.02; −0.8), 2004 (−0.01; −0.82), 2005 (−0.03; −0.78), 2006 (0.11; −0.58), 2007 (0.11; −0.58), 2008 (0.05; −0.57), 2009 (0.06; −0.61), 2010 (0.09; −0.56), 2011 (0.19; −0.42), 2013 (0.23; −0.49), 2015 (0.17; −0.52), 2016 (0.12; −0.61), 2017 (0.16; −0.65), 2018 (0.12; −0.73). Thus, we report correlations based on the raw subjective income data.

The GDP per capita, the top 10% share, and the bottom 50% share of the national income data came from https://wid.world/.

## Supplementary Material

pgac224_Supplemental_File

## Data Availability

All data (aggregated at the country-level) and the analytic codes are available in the Open Science Framework at https://osf.io/x5gcw.
