# Correction to: Money and happiness: the income–happiness correlation is higher when income inequality is higher

**DOI:** 10.1093/pnasnexus/pgad473

**Published:** 2024-01-30

This is a correction to: Shigehiro Oishi, Youngjae Cha, Asuka Komiya, Hiroshi Ono, Money and happiness: the income–happiness correlation is higher when income inequality is higher, *PNAS Nexus*, Volume 1, Issue 5, November 2022, pgac224, https://doi.org/10.1093/pnasnexus/pgac224

During a retroactive audit conducted by *PNAS Nexus*, it was discovered that this paper was missing a statement acknowledging compliance with the *PNAS Nexus* Human and Animal Participants and Clinical Trials policy:

All data analyzed were secondary data. Data analyses were approved by the University of Virginia IRB (Title: Good Life Study; Protocol Number: 3950).

This error has been corrected in the original article.

