# Supplementary material for: Money and happiness: the income–happiness correlation is higher when income inequality is higher
Source: PNAS Nexus. 2022 Oct 8;1(5):pgac224. doi: 10.1093/pnasnexus/pgac224 (PMC9802463; doi:10.1093/pnasnexus/pgac224)
Supplement: pgac224_Supplemental_File [file pgac224_supplemental_file.pdf]

PNAS nexus

Supplementary Information for

Money and Happiness: The Income-Happiness Correlation is Higher When Income Inequality is Higher

Shigehiro Oishi[a], Youngjae Cha[b], Asuka Komiya[c], Hiroshi Ono[d]

a. Department of Psychology, University of Chicago, Chicago, IL

b. Department of Psychology, University of Virginia, Charlottesville, VA

c. Graduate School of Integrated Arts and Sciences, Hiroshima University, Hiroshima, Japan

d. School of International Corporate Strategy, Hitotsubashi University Business School, Tokyo, Japan

*Shigehiro Oishi

Email: soishi@uchicago.edu

This PDF file includes:

Supplementary Material 1
  Tables S1 to S36 (Descriptive Statistics)
Supplementary Material 2
  Figures S1 to S4 (Time trends in GDP across 36 countries)
  Figures S5 to S8 (Time trends in top10% national income share across 36 countries)

Other supplementary materials for this manuscript include the following:

Supplementary Material 3 (Income category values and midpoint values)
  Available at https://osf.io/x5gcw/

Supplementary Material 1

Tables S1-S36 below represent the descriptive statistics and correlations among year, GDP per capita (LN), Top 10%, Bottom50% of each nation. For all the tables, Year2 indicates the survey year recoded to start at zero, the first year of the available survey year for each country (e.g., 1972 for the U.S., 1978 for Japan). zLNGDP indicates *z*-transformed log-transformed GDP per capita. Top10share and Bottom50share indicate the income share held by highest 10% and the lowest 50%. M and SD are used to represent mean and standard deviation, respectively. Values in square brackets indicate the 95% confidence interval for each correlation. The confidence interval is a plausible range of population correlations that could have caused the sample correlation (Cumming, 2014). * indicates $p < .05$. ** indicates $p < .01$.

Table S1: FRANCE

Means, standard deviations, and correlations with confidence intervals

Variable	*M*	*SD*	1	2	3
1. Year2	21.50	14.58			
2. zLNGDP	-0.00	1.00	.96**<br>[.94, .98]		
3. Top10share	0.31	0.02	.15<br>[-.13, .41]	.06<br>[-.23, .33]	
4. Bottom50share	0.22	0.01	-.07<br>[-.34, .22]	.06<br>[-.22, .34]	-.95**<br>[-.97, -.92]

Table S2: BELGIUM

Means, standard deviations, and correlations with confidence intervals

Variable	*M*	*SD*	1	2	3
1. Year2	21.50	14.58			
2. zLNGDP	-0.00	1.00	.99**<br>[.98, .99]		
3. Top10share	0.26	0.01	.16<br>[-.16, .45]	.24<br>[-.08, .51]	
4. Bottom50share	0.27	0.01	.20<br>[-.11, .48]	.12<br>[-.20, .41]	-.73**<br>[-.85, -.54]

Table S3: NETHERLANDS

Means, standard deviations, and correlations with confidence intervals

Variable	*M*	*SD*	1	2	3
1. Year2	21.50	14.58			
2. zLNGDP	-0.00	1.00	.95**<br>[.91, .97]		
3. Top10share	0.23	0.01	.84**<br>[.72, .91]	.88**<br>[.78, .93]	

4. Bottom50share	0.30	0.01	-.88**<br>[-.94, -.78]	-.78**<br>[-.88, -.62]	-.83**<br>[-.91, -.71]

Table S4: GERMANY

Means, standard deviations, and correlations with confidence intervals

Variable	*M*	*SD*	1	2	3
1. Year2	21.50	14.58			
2. zLNGDP	0.00	1.00	.96**<br>[.94, .98]		
3. Top10share	0.32	0.04	.93**<br>[.88, .96]	.90**<br>[.82, .94]	
4. Bottom50share	0.22	0.02	-.96**<br>[-.98, -.93]	-.95**<br>[-.97, -.90]	-.99**<br>[-1.00, -.98]

Table S5: ITALY

Means, standard deviations, and correlations with confidence intervals

Variable	*M*	*SD*	1	2	3
1. Year2	21.50	14.58			
2. zLNGDP	0.00	1.00	.84**<br>[.74, .91]		
3. Top10share	0.28	0.03	.89**<br>[.81, .94]	.75**<br>[.59, .86]	
4. Bottom50share	0.23	0.02	-.91**<br>[-.95, -.84]	-.83**<br>[-.91, -.71]	-.98**<br>[-.99, -.97]

Table S6: LUXEMBOURG

Means, standard deviations, and correlations with confidence intervals

Variable	*M*	*SD*	1	2	3
1. Year2	21.50	14.58			
2. zLNGDP	0.00	1.00	.96**<br>[.94, .98]		
3. Top10share	0.36	0.02	-.29<br>[-.55, .03]	-.01<br>[-.32, .30]	
4. Bottom50share	0.22	0.01	-.80**<br>[-.89, -.64]	-.81**<br>[-.90, -.67]	-.21<br>[-.49, .10]

Table S7: DENMARK

Means, standard deviations, and correlations with confidence intervals

Variable	*M*	*SD*	1	2	3
1. Year2	21.50	14.58			
2. zLNGDP	0.00	1.00	.98**<br>[.97, .99]		
3. Top10share	0.29	0.02	.41**<br>[.15, .62]	.38**<br>[.12, .60]	
4. Bottom50share	0.26	0.01	-.81**<br>[-.89, -.66]	-.67**<br>[-.81, -.46]	-.87**<br>[-.93, -.76]

Table S8: IRELAND

Means, standard deviations, and correlations with confidence intervals

Variable	*M*	*SD*	1	2	3
1. Year2	21.50	14.58			
2. zLNGDP	0.00	1.00	.98**<br>[.97, .99]		
3. Top10share	0.31	0.03	.82**<br>[.70, .90]	.89**<br>[.80, .94]	
4. Bottom50share	0.23	0.01	-.86**<br>[-.92, -.75]	-.85**<br>[-.92, -.72]	-.82**<br>[-.90, -.69]

Table S9: UK

Means, standard deviations, and correlations with confidence intervals

Variable	*M*	*SD*	1	2	3
1. Year2	21.50	14.58			
2. zLNGDP	0.00	1.00	.98**<br>[.96, .99]		
3. Top10share	0.33	0.04	.91**<br>[.85, .95]	.96**<br>[.93, .98]	
4. Bottom50share	0.21	0.01	-.61**<br>[-.77, -.36]	-.75**<br>[-.86, -.57]	-.89**<br>[-.94, -.80]

Table S10: GREECE

Means, standard deviations, and correlations with confidence intervals

Variable	*M*	*SD*	1	2	3
1. Year2	21.50	14.58			
2. zLNGDP	0.00	1.00	.60**<br>[.39, .76]		
3. Top10share	0.36	0.02	-.48**	-.36*	

			[-.67, -.23]	[-.58, -.09]	
4. Bottom50share	0.18	0.01	.44**<br>[.15, .66]	.15<br>[-.17, .44]	-.84**<br>[-.91, -.71]

Table S11: SPAIN

Means, standard deviations, and correlations with confidence intervals

Variable	*M*	*SD*	1	2	3
1. Year2	21.50	14.58			
2. zLNGDP	-0.00	1.00	.97**<br>[.95, .98]		
3. Top10share	0.35	0.01	-.74**<br>[-.86, -.56]	-.75**<br>[-.86, -.57]	
4. Bottom50share	0.21	0.01	.78**<br>[.61, .88]	.72**<br>[.52, .84]	-.90**<br>[-.94, -.81]

Table S12: PORTUGAL

Means, standard deviations, and correlations with confidence intervals

Variable	*M*	*SD*	1	2	3
1. Year2	21.50	14.58			
2. zLNGDP	0.00	1.00	.94**<br>[.90, .97]		
3. Top10share	0.37	0.03	.72**<br>[.52, .84]	.87**<br>[.76, .93]	
4. Bottom50share	0.19	0.02	-.60**<br>[-.77, -.35]	-.75**<br>[-.86, -.57]	-.97**<br>[-.98, -.94]

Table S13: NORWAY

Means, standard deviations, and correlations with confidence intervals

Variable	*M*	*SD*	1	2	3
1. Year2	21.50	14.58			
2. zLNGDP	-0.00	1.00	.97**<br>[.95, .99]		
3. Top10share	0.29	0.03	.30*<br>[.02, .53]	.42**<br>[.17, .63]	
4. Bottom50share	0.28	0.02	-.74**<br>[-.85, -.55]	-.85**<br>[-.92, -.73]	-.95**<br>[-.98, -.91]

Table S14: FINLAND

Means, standard deviations, and correlations with confidence intervals

Variable	*M*	*SD*	1	2	3
1. Year2	21.50	14.58			
2. zLNGDP	-0.00	1.00	.97**<br>[.94, .98]		
3. Top10share	0.29	0.03	.84**<br>[.71, .91]	.89**<br>[.80, .94]	
4. Bottom50share	0.25	0.01	-.86**<br>[-.92, -.75]	-.84**<br>[-.91, -.71]	-.83**<br>[-.91, -.70]

Table S15: SWEDEN

Means, standard deviations, and correlations with confidence intervals

Variable	*M*	*SD*	1	2	3
1. Year2	21.50	14.58			
2. zLNGDP	-0.00	1.00	.98**<br>[.97, .99]		
3. Top10share	0.29	0.02	.39**<br>[.12, .60]	.40**<br>[.14, .61]	
4. Bottom50share	0.27	0.01	-.86**<br>[-.92, -.74]	-.83**<br>[-.91, -.70]	-.79**<br>[-.89, -.64]

Table S16: AUSTRIA

Means, standard deviations, and correlations with confidence intervals

Variable	*M*	*SD*	1	2	3
1. Year2	21.50	14.58			
2. zLNGDP	-0.00	1.00	.98**<br>[.96, .99]		
3. Top10share	0.23	0.01	.69**<br>[.48, .83]	.67**<br>[.45, .81]	
4. Bottom50share	0.33	0.01	.03<br>[-.29, .33]	.16<br>[-.16, .45]	-.45**<br>[-.67, -.17]

Table S17: ARGENTINA

Means, standard deviations, and correlations with confidence intervals

Variable	*M*	*SD*	1	2	3
1. Year2	10.33	7.32			
2. zLNGDP	0.00	1.00	.79**<br>[.53, .92]		
3. Top10share	0.45	0.05	-.74**	-.65**	

Variable	*M*	*SD*	1	2	3
			[-.89, -.45]	[-.85, -.28]	
4. Bottom50share	0.14	0.03	.91**<br>[.77, .96]	.69**<br>[.34, .87]	-.90**<br>[-.96, -.75]

Table S18: BOLIVIA

Means, standard deviations, and correlations with confidence intervals

Variable	*M*	*SD*	1	2	3
1. Year2	10.87	6.99			
2. zLNGDP	-0.00	1.00	.97**<br>[.93, .99]		
3. Top10share	0.51	0.02	-.90**<br>[-.96, -.76]	-.88**<br>[-.95, -.70]	
4. Bottom50share	0.11	0.01	.96**<br>[.90, .98]	.94**<br>[.84, .98]	-.98**<br>[-.99, -.94]

Table S19: BRASIL

Means, standard deviations, and correlations with confidence intervals

Variable	*M*	*SD*	1	2	3
1. Year2	10.33	7.32			
2. zLNGDP	0.00	1.00	.83**<br>[.60, .93]		
3. Top10share	0.56	0.01	.59**<br>[.20, .82]	.61**<br>[.22, .83]	
4. Bottom50share	0.11	0.00	-.51*<br>[-.77, -.08]	-.16<br>[-.57, .32]	-.70**<br>[-.87, -.37]

Table S20: CHILE

Means, standard deviations, and correlations with confidence intervals

Variable	*M*	*SD*	1	2	3
1. Year2	10.33	7.32			
2. zLNGDP	-0.00	1.00	.93**<br>[.81, .97]		
3. Top10share	0.61	0.01	.17<br>[-.30, .57]	.43<br>[-.03, .74]	
4. Bottom50share	0.09	0.01	.50*<br>[.07, .77]	.15<br>[-.33, .57]	-.65**<br>[-.85, -.29]

Table S21: COLOMBIA

Means, standard deviations, and correlations with confidence intervals

Variable	*M*	*SD*	1	2	3
1. Year2	10.87	6.99			
2. zLNGDP	-0.00	1.00	.89**<br>[.72, .96]		
3. Top10share	0.51	0.02	-.87**<br>[-.95, -.70]	-.64**<br>[-.85, -.26]	
4. Bottom50share	0.11	0.01	.91**<br>[.77, .96]	.66**<br>[.29, .86]	-.98**<br>[-.99, -.96]

Table S22: COSTA RICA
Means, standard deviations, and correlations with confidence intervals

Variable	*M*	*SD*	1	2	3
1. Year2	10.87	6.99			
2. zLNGDP	0.00	1.00	.98**<br>[.96, .99]		
3. Top10share	0.49	0.02	.85**<br>[.66, .94]	.83**<br>[.61, .93]	
4. Bottom50share	0.10	0.01	-.87**<br>[-.95, -.70]	-.82**<br>[-.93, -.59]	-.95**<br>[-.98, -.88]

Table S23: DOMINICAN REPUBLIC
Means, standard deviations, and correlations with confidence intervals

Variable	*M*	*SD*	1	2	3
1. Year2	12.50	5.92			
2. zLNGDP	-0.00	1.00	.95**<br>[.87, .98]		
3. Top10share	0.51	0.02	-.90**<br>[-.96, -.76]	-.81**<br>[-.93, -.57]	
4. Bottom50share	0.11	0.01	.96**<br>[.90, .98]	.87**<br>[.69, .95]	-.98**<br>[-.99, -.94]

Table S24: ECUADOR
Means, standard deviations, and correlations with confidence intervals

Variable	*M*	*SD*	1	2	3
1. Year2	10.87	6.99			
2. zLNGDP	0.00	1.00	.96**<br>[.90, .99]		
3. Top10share	0.43	0.04	-.90**	-.78**	

			[-.96, -.77]	[-.91, -.50]	
4. Bottom50share	0.14	0.01	.82**<br>[.59, .93]	.66**<br>[.30, .86]	-.95**<br>[-.98, -.88]

Table S25: EL SALVADOR

Means, standard deviations, and correlations with confidence intervals

Variable	*M*	*SD*	1	2	3
1. Year2	10.87	6.99			
2. zLNGDP	0.00	1.00	.99**<br>[.98, 1.00]		
3. Top10share	0.48	0.03	-.50*<br>[-.77, -.07]	-.36<br>[-.70, .11]	
4. Bottom50share	0.09	0.01	.69**<br>[.36, .87]	.66**<br>[.30, .86]	-.80**<br>[-.92, -.56]

Table S26: GUATEMALA

Means, standard deviations, and correlations with confidence intervals

Variable	*M*	*SD*	1	2	3
1. Year2	10.87	6.99			
2. zLNGDP	0.00	1.00	.99**<br>[.98, 1.00]		
3. Top10share	0.51	0.02	-.90**<br>[-.96, -.76]	-.84**<br>[-.94, -.63]	
4. Bottom50share	0.11	0.01	.96**<br>[.90, .98]	.92**<br>[.81, .97]	-.98**<br>[-.99, -.94]

Table S27: HONDURAS

Means, standard deviations, and correlations with confidence intervals

Variable	*M*	*SD*	1	2	3
1. Year2	10.87	6.99			
2. zLNGDP	-0.00	1.00	.98**<br>[.95, .99]		
3. Top10share	0.51	0.02	-.90**<br>[-.96, -.76]	-.84**<br>[-.93, -.61]	
4. Bottom50share	0.11	0.01	.96**<br>[.90, .98]	.91**<br>[.77, .96]	-.98**<br>[-.99, -.94]

Table S28: MEXICO

Means, standard deviations, and correlations with confidence intervals

Variable	*M*	*SD*	1	2	3
1. Year2	10.33	7.32			
2. zLNGDP	-0.00	1.00	.73**<br>[.41, .89]		
3. Top10share	0.57	0.03	.83**<br>[.62, .93]	.88**<br>[.71, .95]	
4. Bottom50share	0.08	0.00	-.12<br>[-.53, .34]	-.54*<br>[-.80, -.11]	-.55*<br>[-.80, -.14]

Table S29: NICARAGUA

Means, standard deviations, and correlations with confidence intervals

Variable	*M*	*SD*	1	2	3
1. Year2	10.87	6.99			
2. zLNGDP	0.00	1.00	.98**<br>[.96, .99]		
3. Top10share	0.51	0.02	-.90**<br>[-.96, -.76]	-.87**<br>[-.95, -.68]	
4. Bottom50share	0.11	0.01	.96**<br>[.90, .98]	.94**<br>[.84, .98]	-.98**<br>[-.99, -.94]

Table S30: PANAMA

Means, standard deviations, and correlations with confidence intervals

Variable	*M*	*SD*	1	2	3
1. Year2	10.87	6.99			
2. zLNGDP	0.00	1.00	.99**<br>[.98, 1.00]		
3. Top10share	0.51	0.02	-.90**<br>[-.96, -.76]	-.90**<br>[-.96, -.75]	
4. Bottom50share	0.11	0.01	.96**<br>[.90, .98]	.96**<br>[.89, .98]	-.98**<br>[-.99, -.94]

Table S31: PARAGUAY

Means, standard deviations, and correlations with confidence intervals

Variable	*M*	*SD*	1	2	3
1. Year2	10.33	7.32			
2. zLNGDP	0.00	1.00	.93**<br>[.83, .97]		
3. Top10share	0.51	0.02	-.90**	-.77**	

			[-.96, -.76]	[-.91, -.48]	
4. Bottom50share	0.11	0.01	.96**<br>[.90, .98]	.85**<br>[.65, .94]	-.98**<br>[-.99, -.94]

Table S32: PERU

Means, standard deviations, and correlations with confidence intervals

Variable	*M*	*SD*	1	2	3
1. Year2	10.33	7.32			
2. zLNGDP	0.00	1.00	.96**<br>[.90, .99]		
3. Top10share	0.56	0.03	-.74**<br>[-.89, -.44]	-.78**<br>[-.91, -.51]	
4. Bottom50share	0.09	0.01	.88**<br>[.72, .95]	.92**<br>[.79, .97]	-.95**<br>[-.98, -.87]

Table S33: URUGUAY

Means, standard deviations, and correlations with confidence intervals

Variable	*M*	*SD*	1	2	3
1. Year2	10.33	7.32			
2. zLNGDP	0.00	1.00	.91**<br>[.78, .97]		
3. Top10share	0.45	0.04	-.74**<br>[-.89, -.44]	-.85**<br>[-.94, -.64]	
4. Bottom50share	0.16	0.02	.82**<br>[.60, .93]	.90**<br>[.75, .96]	-.97**<br>[-.99, -.94]

Table S34: VENEZUELA

Means, standard deviations, and correlations with confidence intervals

Variable	*M*	*SD*	1	2	3
1. Year2	10.33	7.32			
2. zLNGDP	0.00	1.00	.65**<br>[.27, .85]		
3. Top10share	0.51	0.02	-.90**<br>[-.96, -.76]	-.43<br>[-.74, .03]	
4. Bottom50share	0.11	0.01	.96**<br>[.90, .98]	.53*<br>[.10, .79]	-.98**<br>[-.99, -.94]

Table S35: JAPAN

Means, standard deviations, and correlations with confidence intervals

Variable	*M*	*SD*	1	2	3
1. Year2	16.50	9.96			
2. zLNGDP	-0.00	1.00	.83**<br>[.64, .93]		
3. Top10share	0.39	0.03	.90**<br>[.80, .95]	.66**<br>[.35, .84]	
4. Bottom50share	0.19	0.01	-.91**<br>[-.96, -.83]	-.63**<br>[-.83, -.28]	-.99**<br>[-1.00, -.98]

Table S36: UNITED STATES

Means, standard deviations, and correlations with confidence intervals

Variable	*M*	*SD*	1	2	3
1. Year2	23.00	13.71			
2. zLNGDP	-0.00	1.00	.98**<br>[.97, .99]		
3. Top10share	0.40	0.04	.98**<br>[.97, .99]	.99**<br>[.98, .99]	
4. Bottom50share	0.16	0.03	-.97**<br>[-.99, -.95]	-.97**<br>[-.98, -.95]	-.98**<br>[-.99, -.97]

Figures S1-S4. Time trends in GDP across 36 countries. For all the figures, the X-axis is the first survey year available in each country. The Y-axis indicates GDPpc capita (log-transformed, standardized). The blue line depicts the total trend. For the detailed correlation coefficient, please see Supplementary Material 1.

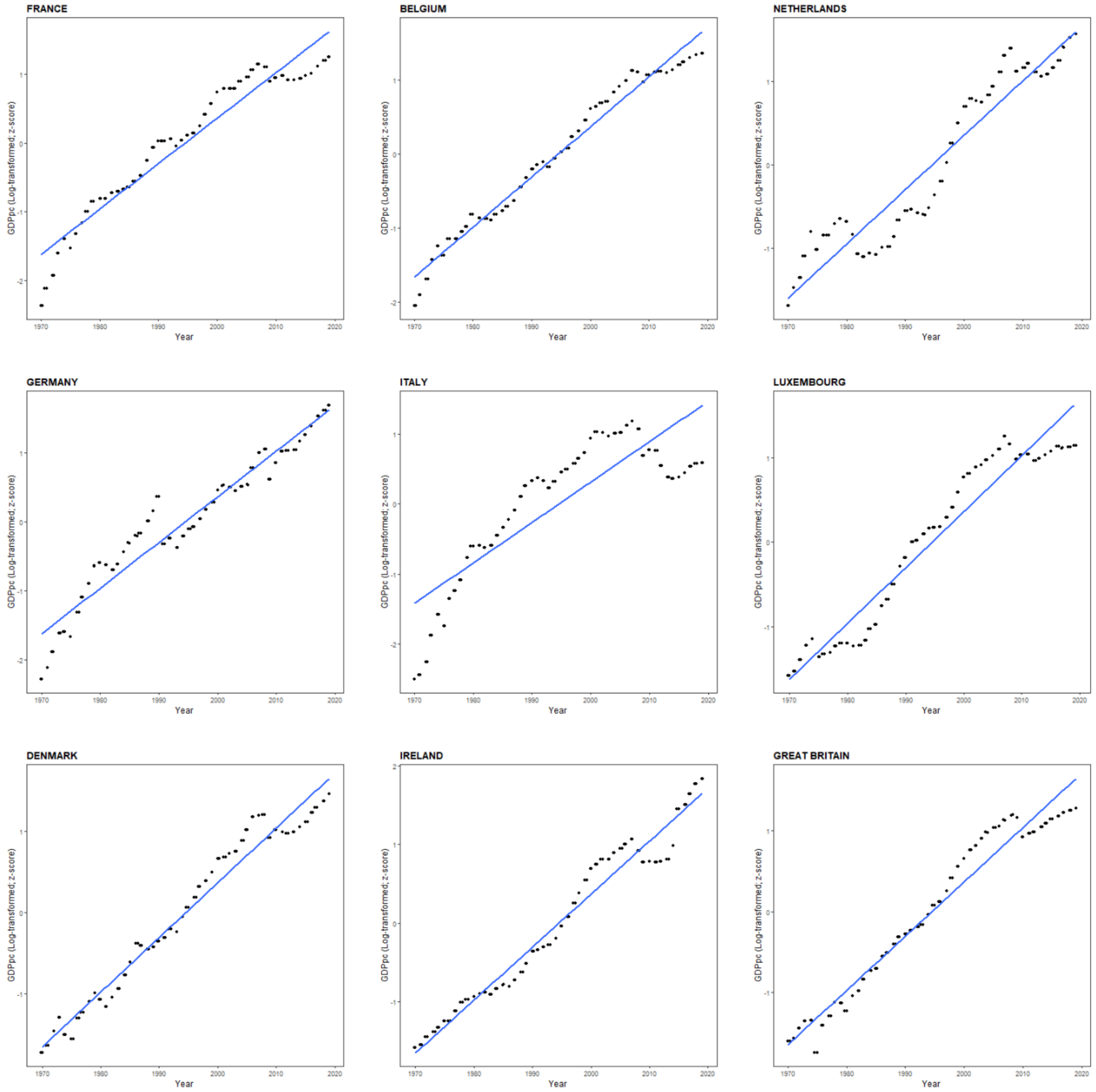

Figure S1. Time trends in GDP in France, Belgium, Netherland, Germany, Italy, Luxembourg, Denmark, Ireland, and UK.

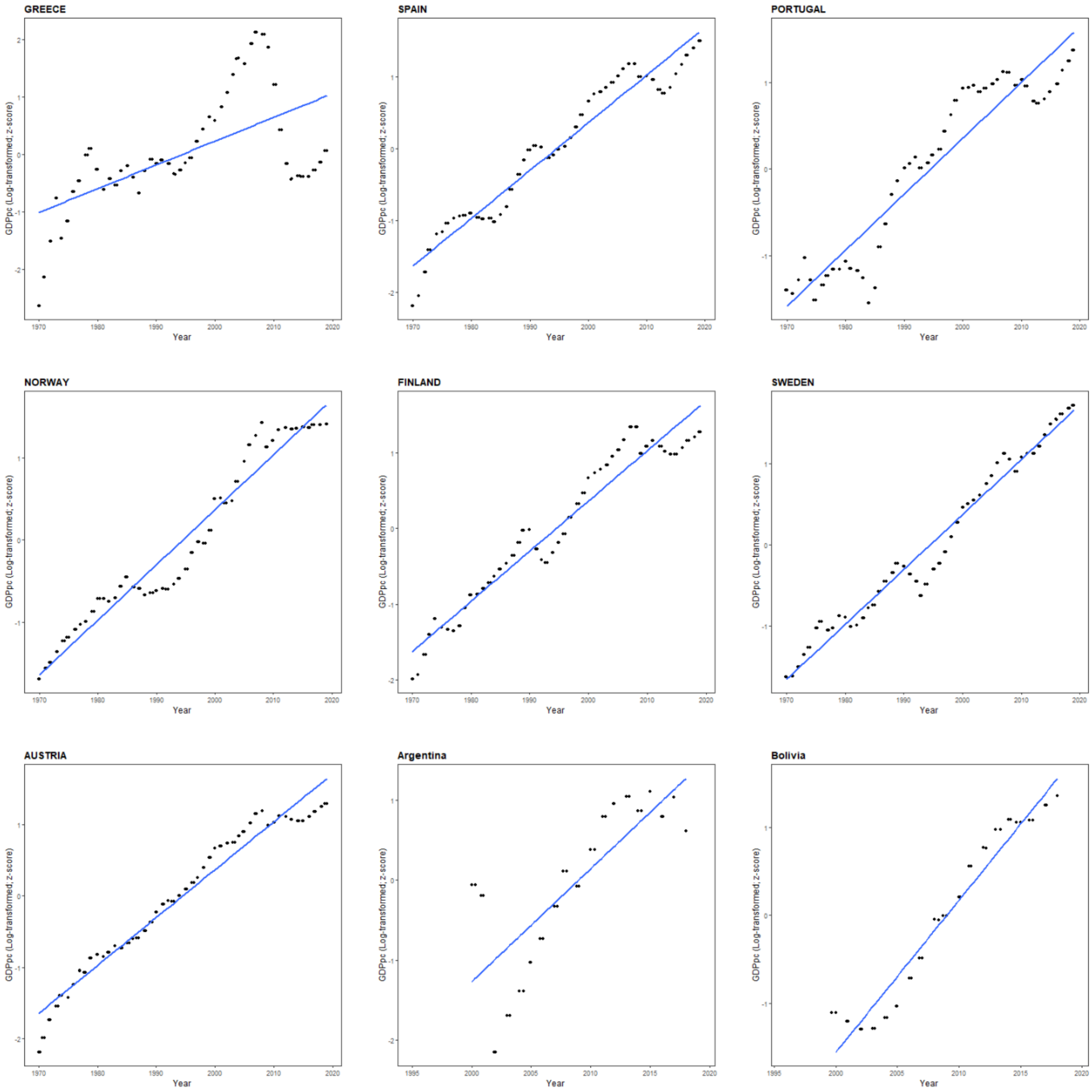

Figure S2. Time trends in GDP in Greece, Spain, Portugal, Finland, Norway, Sweden, Austria, Argentina, and Bolivia

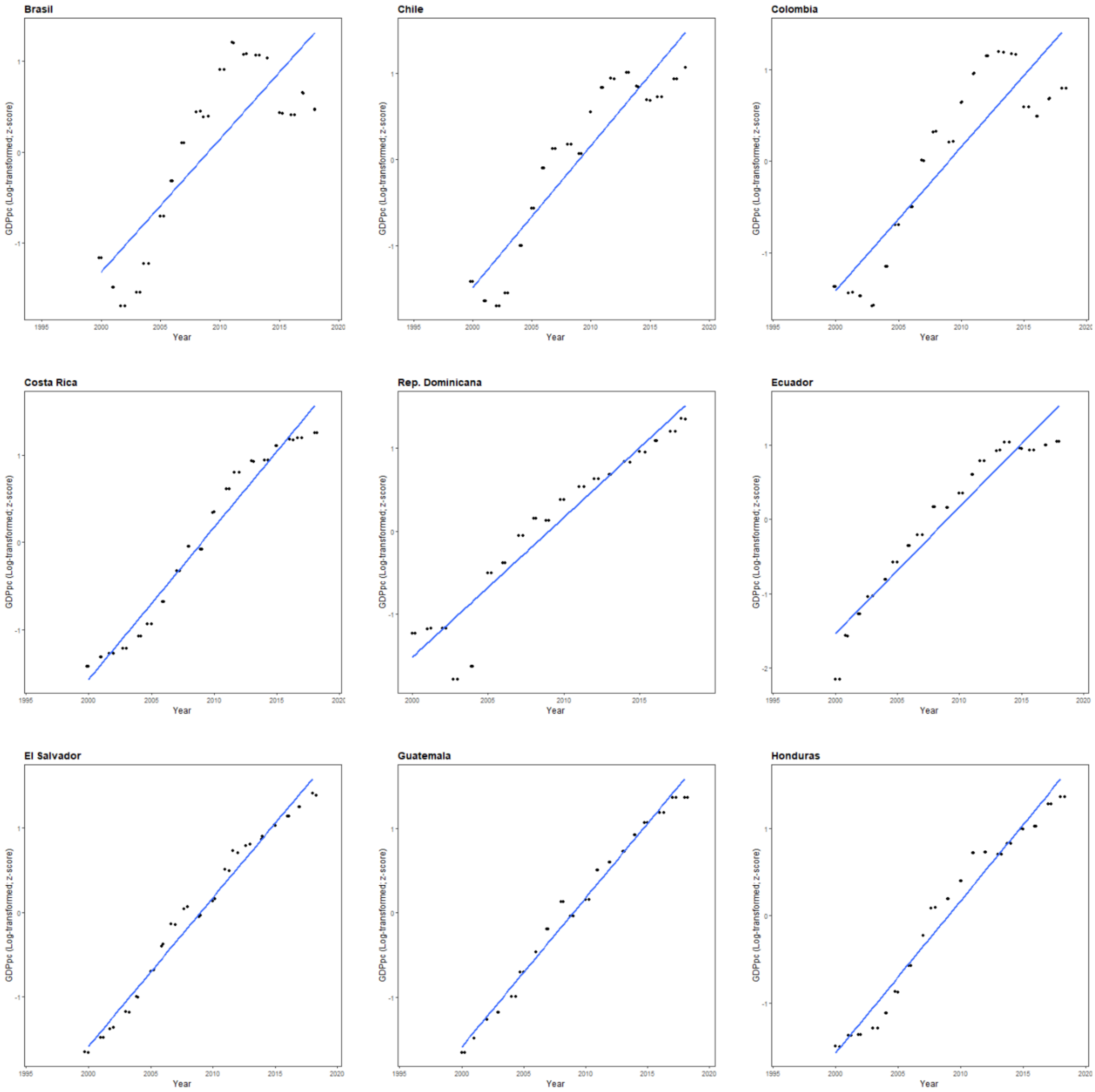

Figure S3. Time trends in GDP in Brasil, Chile, Colombia, Costa Rica, Rep. Dominicana, Ecuador, El Salvador, Guatemala, and Honduras

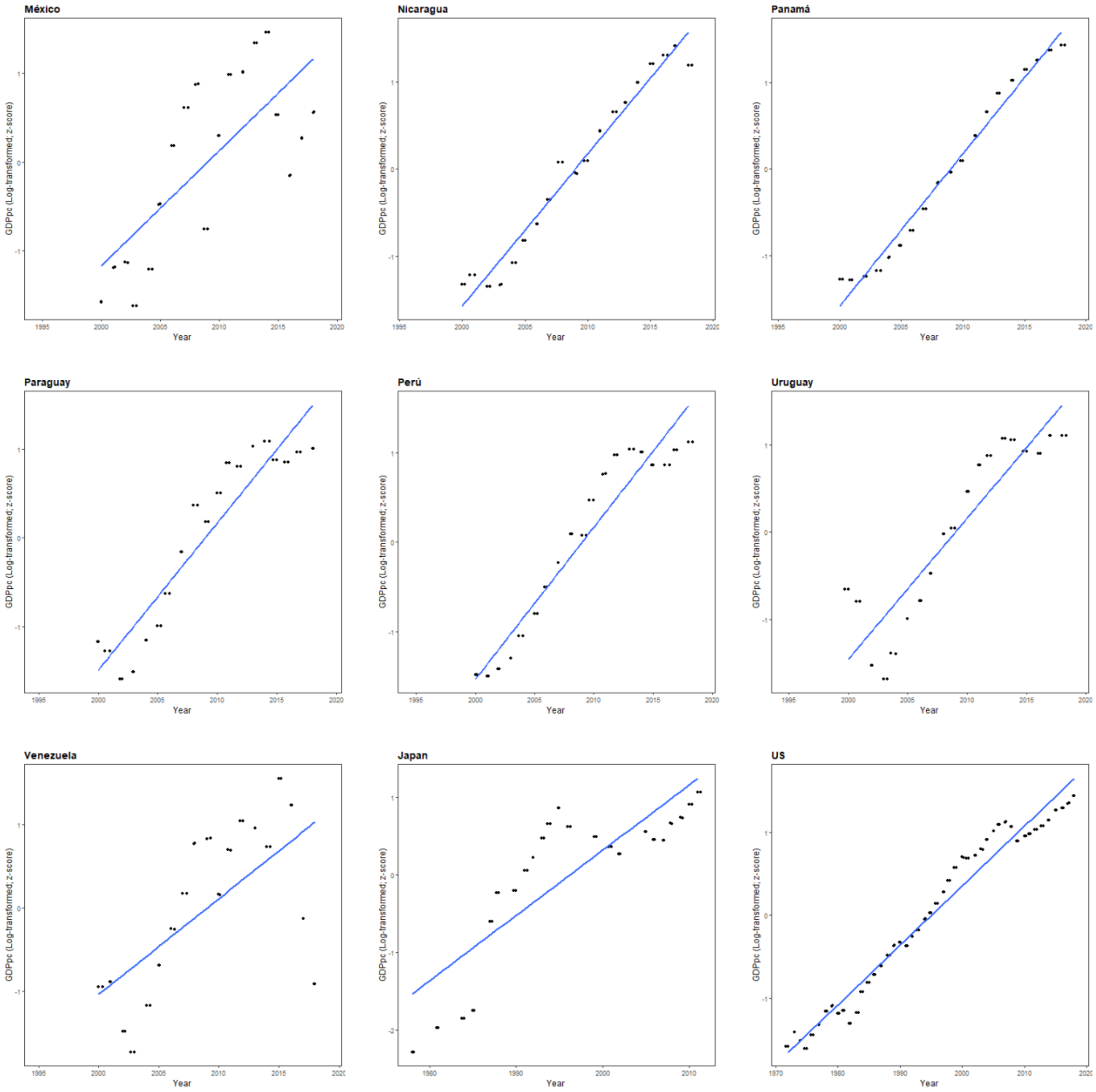

Figure S4. *Time trends in GDP in* México, Nicaragua, Panamá, Paraguay, Perú, Uruguay, Venezuela, Japan, US

Figures S5-S8. Time trends in Top10%Share across 36 countries. For all the figures, the X-axis is the first survey year available in each country. The Y-axis indicates the income share by Top 10% earners in a country. The blue line depicts the total trend.

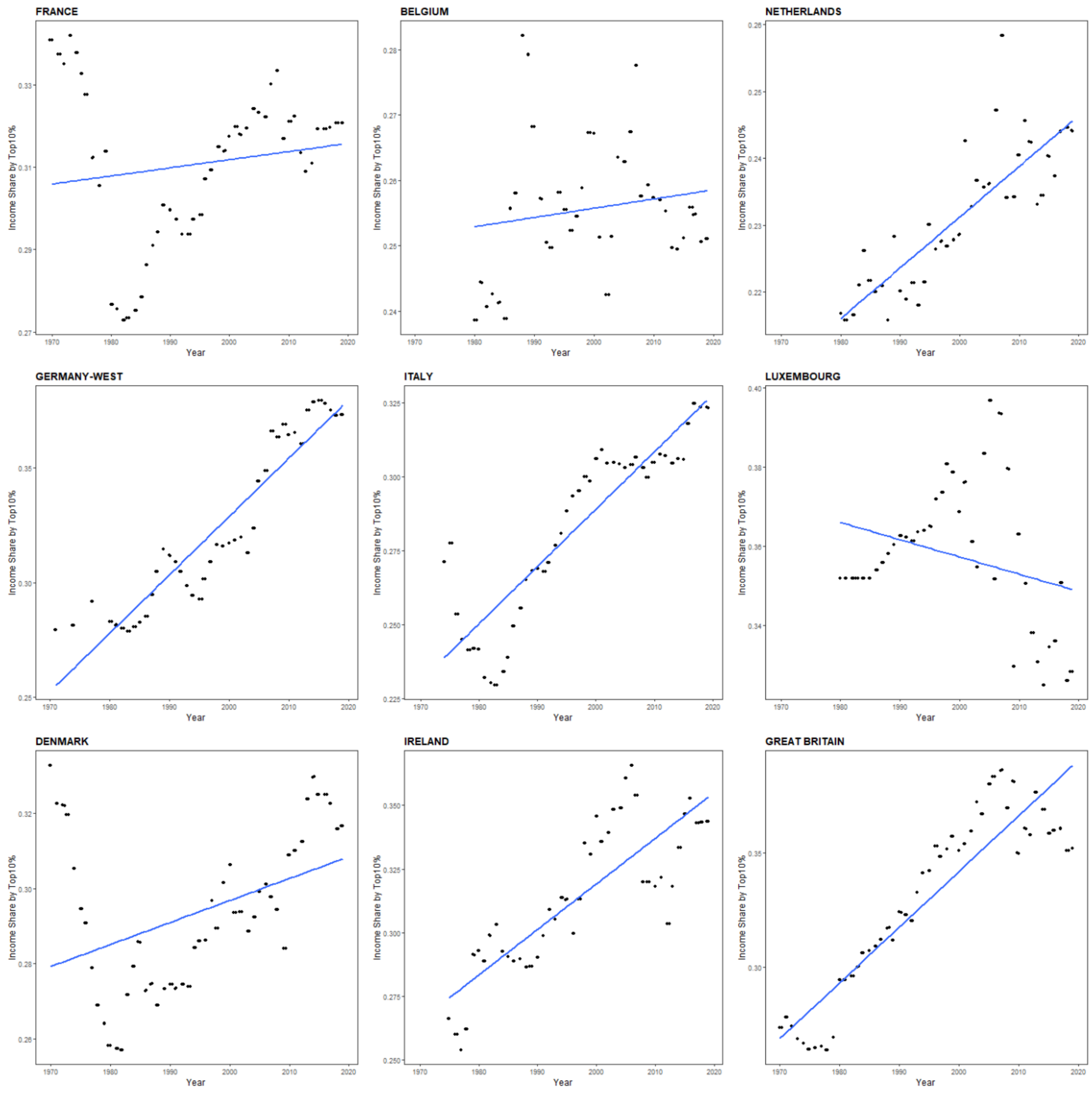

Figure S5. Time trends in Top10% Share in France, Belgium, Netherland, Germany, Italy, Luxembourg, Denmark, Ireland, and UK.

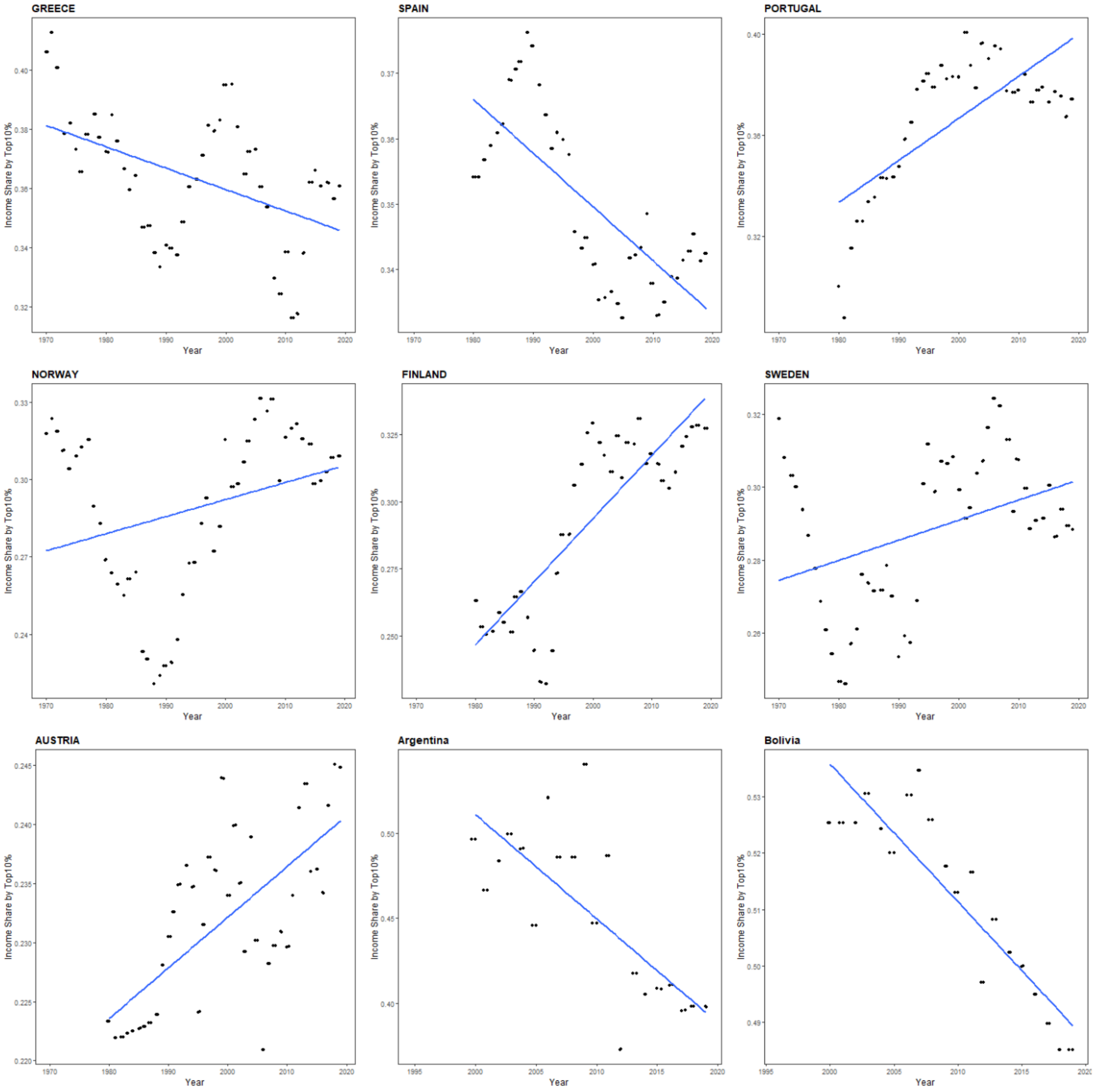

Figure S6. Time trends in Top10% Share in Greece, Spain, Portugal, Finland, Norway, Sweden, Austria, Argentina, and Bolivia

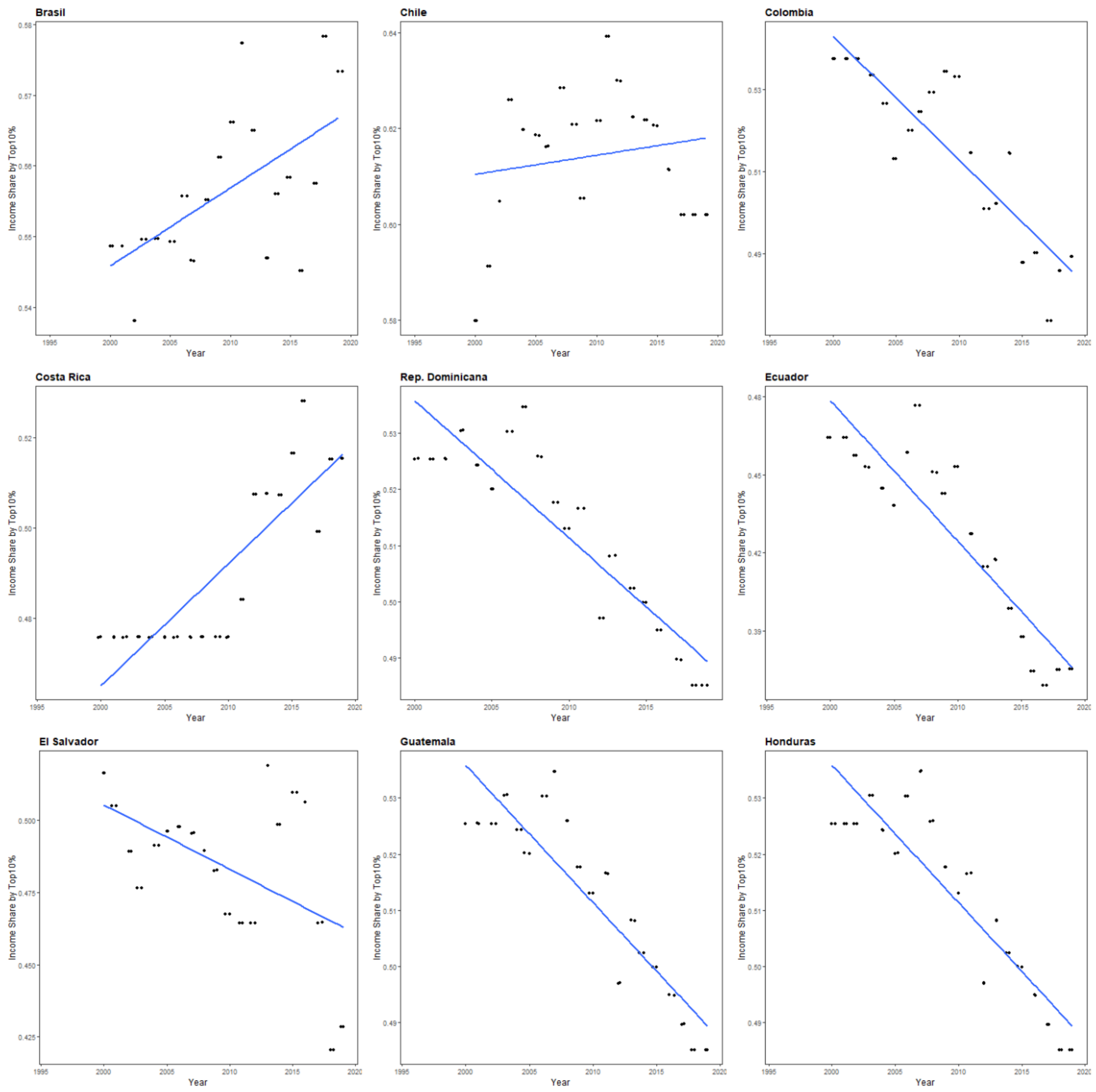

Figure S7. *Time trends in Top 10% Share in Brasil, Chile, Colombia, Costa Rica, Rep. Dominicana, Ecuador, El Salvador, Guatemala, and Honduras*

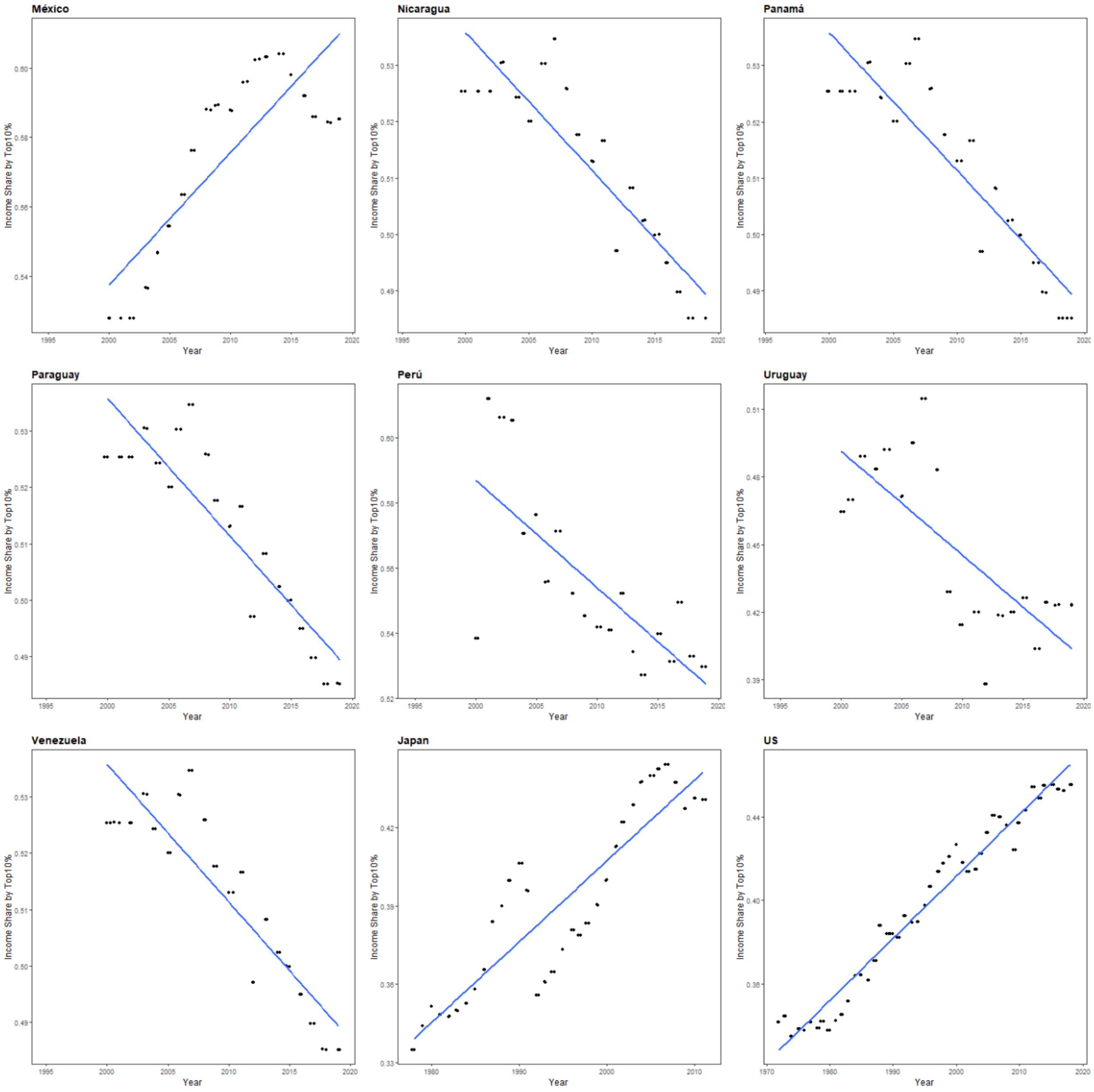

Figure S8. *Time trends in Top 10% Share in* México, Nicaragua, Panamá, Paraguay, Perú, Uruguay, Venezuela, Japan, US
